# Efficient and Transferable Machine Learning Potentials for Zn-Coordinated Zeolitic Imidazolate Frameworks Through Structural Diversity Sampling and Transfer Learning

**DOI:** 10.1021/acs.jctc.6c01271

**Published:** 2026-09-30

**Authors:** Shang-Wei Lin, Yen-Yung Wu, Li-Chiang Lin

**Affiliations:** Department of Chemical Engineering, 33561 National Taiwan University, Taipei 10617, Taiwan

## Abstract

Zeolitic Imidazolate Frameworks (ZIFs) exhibit remarkable structural flexibility that governs many of their functional properties, yet accurately describing their dynamic behavior remains a significant computational challenge. Classical force fields often fail to capture framework flexibility, whereas first-principles methods are prohibitively expensive. In this work, high-fidelity machine learning potentials (MLPs) are first developed for ZIF-8 using the equivariant Allegro architecture. To reduce the cost associated with the development of MLPs, an active-learning-inspired structural selection strategy based on the smooth overlap of atomic positions (SOAP) is implemented, allowing DFT-level accuracy to be achieved with fewer training configurations. Benchmarking against currently available models demonstrates that, while those models also offer reasonable energy predictions, their force errors remain 1 order of magnitude larger than the specialized MLP developed herein with a force mean absolute error of as small as 0.016 eV/Å. Building upon these findings, a transferable MLP spanning diverse Zn-based ZIFs is further constructed. Moreover, a hierarchical transfer learning workflow is also established to enable rapid adaptation to unseen topologies with improved accuracy using only a limited number of additional configurations. As a demonstration, MD simulations powered by the developed MLP successfully capture methane-induced gate-opening in ZIF-8, resolving the structural transition at subnanosecond resolution. Overall, this work provides a transferable, DFT-accurate machine learning force field for Zn-based ZIFs.

## Graphical abstract

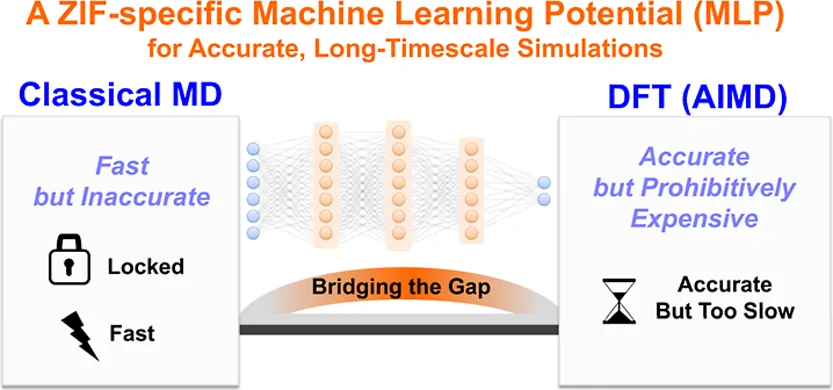

## Introduction

Metal–organic frameworks (MOFs) have emerged as a revolutionary class of crystalline coordination polymers, characterized by their exceptional porosity, vast surface area, and nearly limitless chemical tunability.^1–3^ Through the principles of reticular chemistry, inorganic nodes and organic linkers can be modularly assembled to form architectures tailored for applications ranging from carbon capture and heterogeneous catalysis to molecular sensing, drug delivery, water adsorption, and encapsulation.^4–10^ Among the expansive MOF landscape, zeolitic imidazolate frameworks (ZIFs) occupy a distinguished position. By mimicking the tetrahedral coordination and bond angles found in zeolites, ZIFs combine the structural diversity of MOFs with exceptional chemical and thermal stability.^3,6^ These characteristics collectively render ZIFs benchmark materials for industrial-scale gas separation and membrane technologies.^11,12^

A defining physical attribute of ZIF frameworks is their intrinsic lattice flexibility, a property that is often central to their functional performance.^11^ This flexibility is most prominently manifested in the gate-opening effect,^13,14^ a structural transition where imidazolate linkers undergo swinging motions to allow passage of guest molecules that are larger than the framework’s nominal pore window.^15,16^ While this dynamic behavior is essential for selective gas permeation, accurately modeling the complex and highly anharmonic potential energy surface (PES) that governs these transitions remains a challenge in the field of computational chemistry.^13,17,18^ The fundamental difficulty lies in the need to resolve subtle many–body interactions within the metal-coordination sphere while simultaneously accounting for the structural deformations of the organic linkers over the hundreds-of-picoseconds-to-nanosecond time scales characteristic of gate-opening dynamics.^19,20^

The dynamic modeling of ZIFs has historically been dominated by classical molecular dynamics (MD) simulations employing force fields such as the universal force field for metal–organic frameworks (UFF4MOF) or AMBER-based potentials.^21–23^ These methods offer high computational efficiency but are fundamentally constrained by their approximation. Specifically, in these classical schemes, the coordination environment is typically described by simple harmonic potentials that cannot account for electronic redistributions during structural fluctuations. Consequently, classical force fields often struggle to accurately capture the framework flexibility and guest-induced structural responses associated with gate-opening phenomena in ZIFs.^24^ Alternatively, reactive force fields (ReaxFFs), including recently developed ZIF-specific parametrizations for Zn–imidazolate systems, enable a more flexible representation of atomic interactions through a bond-order-dependent formalism and have recently been applied to investigate the stability and reactivity of ZIF materials.^25,26^ However, the application of ReaxFFs to multielement systems remains challenging. Particularly, the complexity of its parametrization often leads to limited transferability across different topologies and chemical environments.^27^ Collectively, achieving the accuracy required to describe the subtle energetic differences associated with ZIF flexibility remains difficult.

While *ab initio* molecular dynamics (AIMD) based on density functional theory (DFT) can ultimately offer the requisite accuracy to capture these subtle interactions that cannot be achieved with the above-mentioned potentials, its cubic scaling with system size renders it computationally prohibitive for the sufficiently long trajectories needed to sample rare structural transitions.^17,28^ Recently, machine learning potentials (MLPs) have emerged as a promising alternative, offering near-DFT accuracy while enabling simulations at substantially larger spatial and temporal scales.^29–31^ A variety of MLP architectures have been developed in recent years, including graph neural network and equivariant architectures such as CHGNet, MACE, ORB, and Allegro, which have demonstrated remarkable success in describing diverse classes of materials.^32–35^ Among these approaches, equivariant architectures have attracted particular attention because their explicit treatment of E(3) symmetry improves both data efficiency and model transferability. The Allegro framework further combines E(3)-equivariant representations with a strictly local architecture, enabling efficient large-scale simulations while maintaining an accurate description of complex atomic interactions.^35^ These characteristics are particularly attractive for flexible ZIF systems, given capturing rare events such as linker swinging or gate-opening transitions requires simulations over extended spatial and temporal scales. However, the development of robust and transferable MLPs remains fundamentally limited by the cost of generating large quantities of first-principles reference data. Moreover, this challenge becomes increasingly severe when extending the model beyond a single framework to encompass diverse topologies, linker chemistries, and thermally activated structural distortions. Recent studies have begun to address these challenges through data-efficient potential construction, transfer learning, and large-scale data sets for porous materials. For example, model-in-the-loop active-learning strategies such as DEAL iteratively identify informative configurations based on model uncertainty,^36^ while related approaches have been developed for constructing and validating machine-learning potentials for MOFs.^29,37^ Large-scale efforts such as ODAC have further expanded the availability of first-principles porous-material data sets.^38^ More recently, fine-tuning of foundation potentials across representative MOF data sets has demonstrated improved transferability for framework-specific properties,^39^ while MOFSimBench has highlighted both the potential and current limitations of universal MLPs for molecular simulations of MOFs.^40^ Collectively, these developments provide increasingly powerful routes toward broadly transferable MOF potentials, but efficient adaptation to a specific chemical environment using only a very limited amount of additional first-principles data remains an important practical challenge.

Herein, efficient and transferable MLPs for Zn-coordinated ZIFs developed through a systematic workflow are reported. To reduce the computational burden associated with their development, we implement a single-shot, model-independent structural sampling strategy based on the smooth overlap of atomic positions (SOAP) descriptor, enabling the identification of structurally representative configurations from a large configurational pool.^41^ This approach improves data efficiency while preserving the essential coordination physics of the Zn-imidazolate network. Building upon this, a pretrained MLP is further developed, which is demonstrated to be transferable to a wide variety of ZIFs. Furthermore, with a hierarchical transfer learning, this foundational MLP can be further refined using very limited target-specific data, enabling improved accuracy for targeted ZIFs. Rather than introducing a new MLP architecture or active-learning algorithm, the present workflow integrates model-independent diversity selection, cross-ZIF representation learning, and data-efficient target adaptation. Finally, the utility of the proposed workflow is demonstrated through the development of an MLP incorporating methane–framework interactions, and MD simulations powered by the MLP successfully capture methane-induced gate-opening in ZIF-8.

## Method

### DFT Reference Data Generation

First-principles reference data are generated using the CP2K software package.^42^ Specifically, electronic structure calculations are performed using the Quickstep module with the PBE exchange–correlation functional. To capture dispersion interactions, the DFT-D3­(BJ) is applied. This study employs a double-ζ valence polarized (DZVP-MOLOPT-SR-GTH) basis set and Goedecker-Teter-Hutter (GTH) pseudopotentials.^43^ The configuration space is sampled through AIMD in the NVT ensemble using a canonical sampling through velocity rescaling thermostat. To ensure a robust description of the potential energy surface, configurations are collected over an extensive temperature range from 300 to 2000 K. This wide temperature window is essential to sample not only energetically favorable states but also the high-energy structural distortions, including bond stretching and linker rotations. Importantly, analysis of the high-temperature trajectories confirms that the Zn–N coordination environment remains largely intact even at the highest temperatures, indicating that these configurations primarily represent thermally distorted rather than decomposed framework structures. More details can be found in the Supporting Information (SI).

### MLP Architecture: Allegro

MLPs reported herein are constructed using the Allegro framework (version 0.7.0), an equivariant neural network designed for high-precision atomic interactions.^35^ Allegro’s strictly local architecture ensures excellent scalability for large-scale simulations while preserving the directional nature of coordination bonds through equivariant tensor features. A radial cutoff of 6.5 Å is adopted to encompass the coordination environment beyond the primary Zn–N bonds, effectively including crucial long-range Zn–Zn correlations. The architecture consists of two equivariant layers with a maximum angular momentum of *l*_max_ = 1, 128 scalar features, and 32 tensor features. The SiLU activation function is employed for all nonlinear mappings. Model optimization is performed using the Adam algorithm with an initial learning rate of 0.001. To ensure a highly converged state and reach the global minimum of the loss function, we implement a dynamic adjustment strategy where the learning rate is automatically reduced whenever the validation error stops improving for a specified period. The loss function is defined as a weighted sum of per-atom energy and force errors, and TF32 precision is utilized to accelerate computations on GPU hardware. Additional details can be found in the SI.

### Structural Sampling and SOAP Selection Strategy

To optimize training efficiency, an active-learning-inspired structural selection workflow is adopted. This strategy utilizes the above-mentioned SOAP descriptor to map local atomic environments into a high-dimensional feature space, followed by a farthest point sampling (FPS) algorithm to identify the most representative configurations.^41,44^ Specifically, the atomic environments are encoded using the SOAP descriptor with a radial cutoff of 6.0 Å. The expansion of the local density is truncated at four for both the maximum radial basis and angular momentum. To represent each multiatom configuration as a single feature vector for comparison, this work employs the inner averaging scheme. This approach provides a global structural fingerprint while maintaining sensitivity to the local coordination chemistry of the Zn nodes and organic linkers. The selection process is conducted through an iterative greedy FPS algorithm. From the initial configuration pool, a starting snapshot is selected randomly. Subsequent frames are chosen by maximizing the minimum Euclidean distance to the set of already selected points in the SOAP feature space. This ensures that the algorithm iteratively selects configurations that are most dissimilar to those already present in the training set. More details can be found in the SI.

### MD Simulations

MLPs developed in this study are integrated into the LAMMPS package for MD simulations.^44,45^ To investigate guest-induced framework flexibility, a methane-loaded ZIF-8 system is constructed by introducing seven methane molecules into the periodic simulation cell. This loading condition is selected to enhance guest–framework interactions and facilitate observation of methane-induced framework flexibility. MD simulations are performed using a time step of 0.5 fs. Temperature and pressure are controlled using the Nose–Hoover thermostat and barostat. Previous experimental adsorption isotherms have shown that gate-opening-related adsorption transitions in ZIF-8 become substantially more pronounced at lower temperatures, whereas the transition gradually diminishes near ambient conditions.^24^ MLP-powered MD simulations are thus performed herein at 200 K and 1 bar. Following an initial equilibration stage, production simulations are conducted to investigate methane motion and framework flexibility. Structural descriptors, including methane–window distances and linker rotation angles, are monitored throughout the MD trajectory to characterize the gate-opening process.

## Results and Discussion

This section discusses the development and evaluation of MLPs for a series of Zn-coordinated ZIF structures with diverse topologies and functional groups, including ZIF-8 in three different sodalite, dia, and kat topologies, as well as ZIF-7, ZIF-11, ZIF-71, ZIF-72, and ZIF-90 (Figure S1). In this study, we first utilize ZIF-8 as a prototypical model to validate the accuracy and efficiency of the developed Allegro-based MLP and benchmark its performance against state-of-the-art MLPs, reactive force fields, and classical force fields. Building upon this baseline and beyond ZIF-8, the training set is further expanded to include ZIF-7, ZIF-71, and ZIF-90. The transferability of the resulting MLP is then evaluated on unseen ZIFs, including alternative ZIF-8 topologies (dia and kat), ZIF-11, and ZIF-72. We further implement a hierarchical transfer-learning workflow to enable rapid adaptation to unseen but targeted systems using only a limited number of additional configurations. Finally, as a demonstration, MLP-powered MD simulations are carried out, which successfully capture gas-induced framework deformation. Note that, for such purpose, methane-containing ZIF-8 configurations are additionally incorporated into the training data set to construct a methane-loaded ZIF-8 MLP capable of describing both framework flexibility and methane–framework interactions within a unified potential.

### Development and Validation of the ZIF-8 Potential

The development of a high-fidelity and transferable MLP for the broader Zn-coordinated ZIF family begins with the construction of a specialized Allegro-based potential for the baseline ZIF-8 system using 40,000 DFT configurations sampled between 300 and 2000 K. The resulting model achieves exceptional predictive accuracy. Specifically, the converged potential yields a per-atom energy MAE of 0.11 meV and an atomic force MAE of 0.016 eV/Å. The corresponding parity plots are presented in Figure 1a, while a quantitative summary of the prediction errors is provided in Figure 2 and Table S1. As can be clearly seen from Figure 1a, both the energy and force parity plots exhibit a near-perfect alignment along the ideal diagonal, indicating its ability to reproduce the potential energy surface with first-principles fidelity. It is important to note that all energy error values reported in this study are calculated after accounting for the systematic absolute energy offsets inherent to different machine learning architectures. In practice, MLPs frequently exhibit a constant energy shift relative to ground-truth values due to differences in the definitions of atomic reference energies. Energy alignment therefore allows comparison of relative energy differences and the detailed corrugation of the potential energy surface associated with the Zn-coordinated environment. For a stricter predictive evaluation, validation-derived energy offsets are additionally applied to the test sets, with the corresponding results provided in the SI.

**Figure 1:**
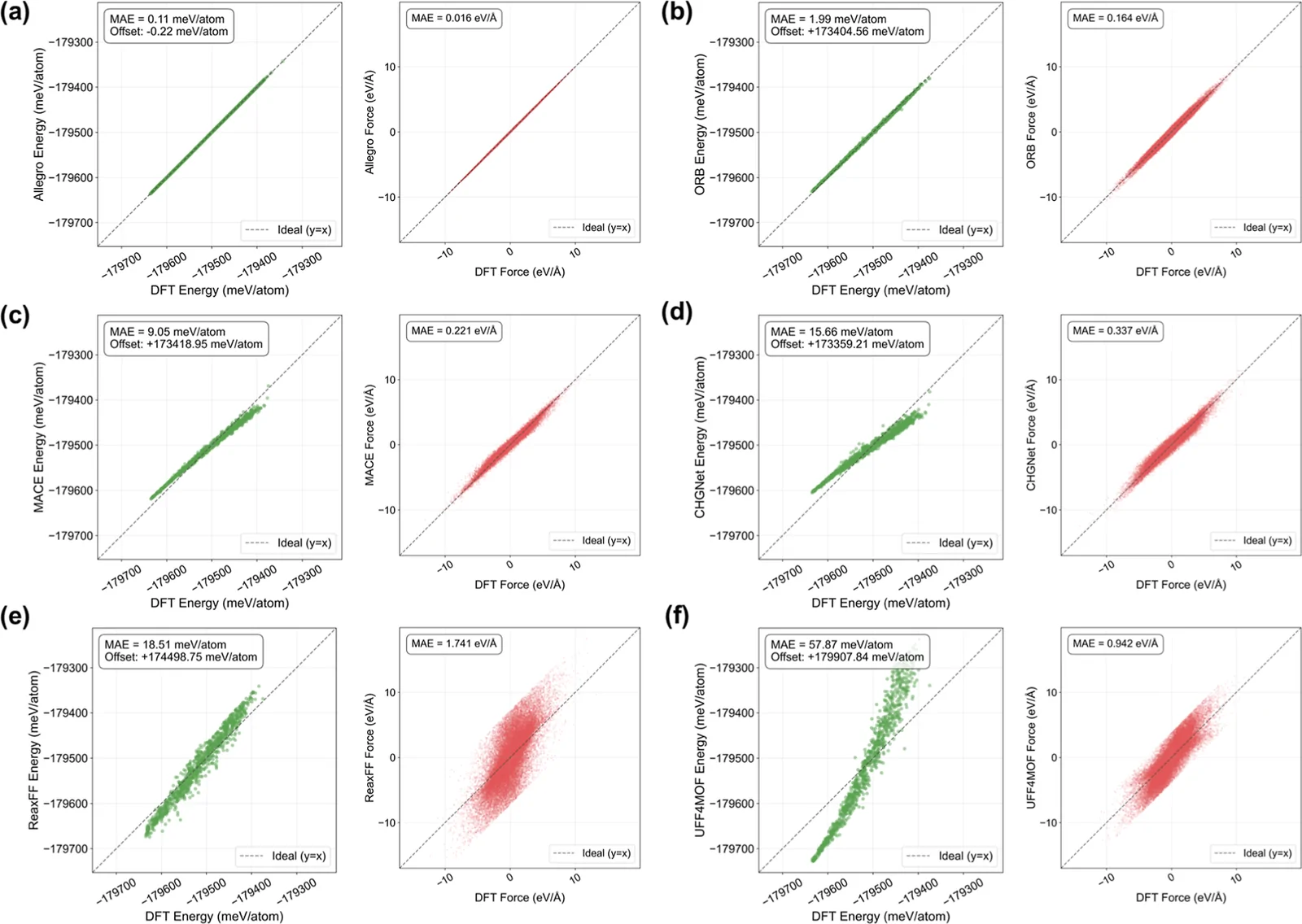
Performance benchmarking of the specialized Allegro MLP developed herein against general-purpose foundation models, reactive potentials, and classical force fields for the ZIF-8 system. Parity plots compare the predicted (MLP/FF) values against the ground-truth DFT reference for total energy (left panel, in meV/atom) and atomic force components (right panel, in eV/Å). The compared models include: (a) our Allegro-based MLP potential, (b) ORB, (c) MACE, (d) CHGNet, (e) ReaxFF, and (f) UFF4MOF.

**Figure 2:**
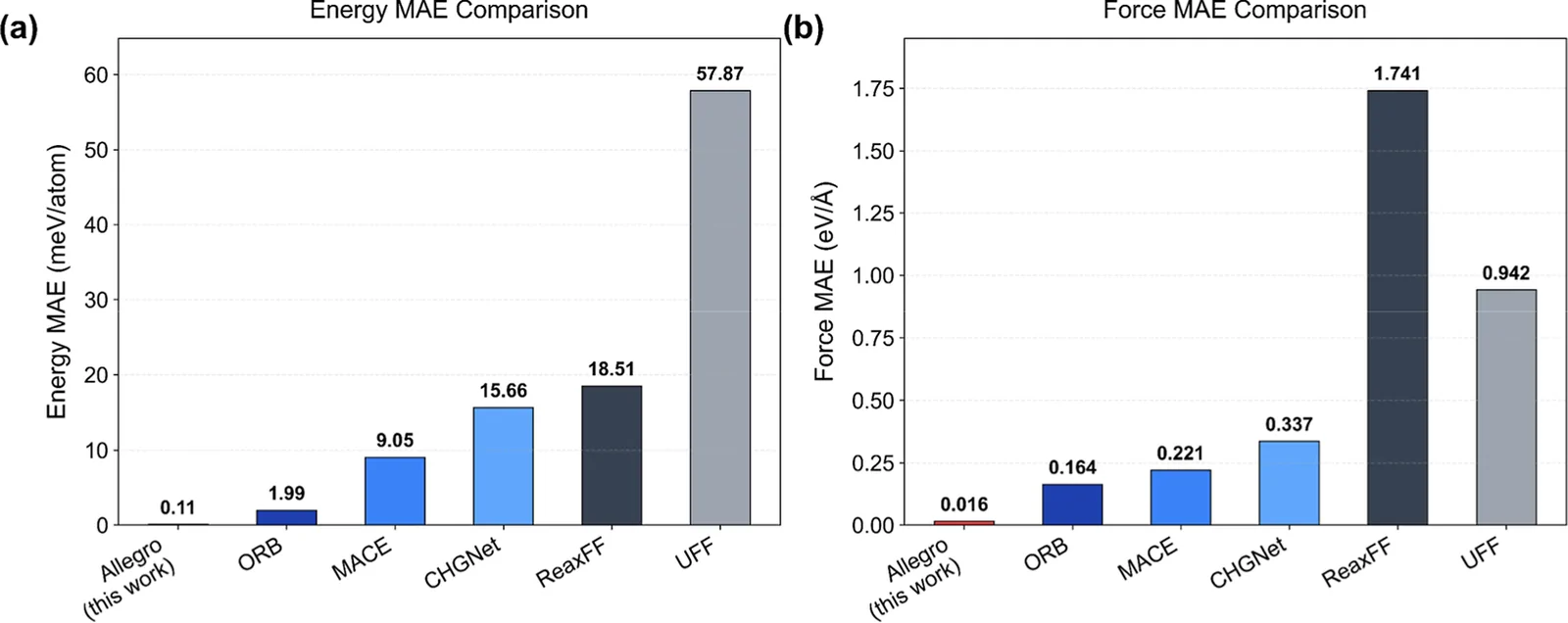
Comparison of per-atom (a) energy MAEs and (b) atomic force MAEs for the Allegro MLP, ORB, MACE, CHGNet, ReaxFF, and UFF4MOF.

To contextualize the performance of the Allegro model, we further compare its accuracy against several state-of-the-art foundation MLPs, including ORB, MACE, and CHGNet. Following the same energy-shifting procedure, ORB, MACE, and CHGNet exhibit varying degrees of energy accuracy, as summarized in Figure 2, with per-atom energy MAEs of 1.99 meV for ORB, 9.05 meV for MACE, and 15.66 meV for CHGNet. Notably, a more significant divergence is observed in the prediction of atomic forces; ORB, MACE, and CHGNet produce force MAEs of 0.164 eV/Å, 0.221 eV/Å, and 0.337 eV/Å, respectively. In comparison, the Allegro-based MLP maintains substantially lower force errors, representing an approximately one-order-of-magnitude improvement relative to these general-purpose architectures. Moreover, Table S2 shows that, compared with MACE-MP-MOF0, the most accurate foundation model benchmarked in this work, the Allegro potential achieves an approximately 5.8-fold lower force MAE (0.016 vs 0.092 eV/Å), while delivering approximately 4.3-fold higher MD throughput while requiring 71% less GPU memory. Together, these results demonstrate that a specialized MLP provides both high predictive fidelity and excellent computational efficiency for ZIF simulations. In this study, we also compare the performance against widely used reactive and classical force fields. As shown in Figure 2, the reactive force field (ReaxFF)^25^ yields a significantly higher force error of 1.741 eV/Å, while UFF4MOF exhibits a force error of 0.942 eV/Å. Both approaches show nearly 2 orders of magnitude larger errors than the MLP developed in this study. These results again highlight the challenges of describing framework flexibility and local coordination environments in ZIF materials using conventional force-field formalisms. Note that, for these models, their parity plots in both the energy and force can be visually seen in Figure 1. Additional finite-temperature validation against CP2K AIMD shows that the MLP reproduces the characteristic Zn–N and Zn–Zn distances and 4-fold Zn coordination of ZIF-8 (Figure S2).

### Improved Training Efficiency via SOAP Sampling

Despite the superior accuracy of the 40,000-snapshot baseline, constructing representative training data sets for multiple ZIF frameworks remains a practical challenge. We therefore investigate whether diversity-aware structural selection can improve data efficiency relative to conventional random sampling. The physical basis for this efficiency is illustrated in Figures 3 and S3, which analyzes the structural diversity and force distribution of the selected training sets.

**Figure 3:**
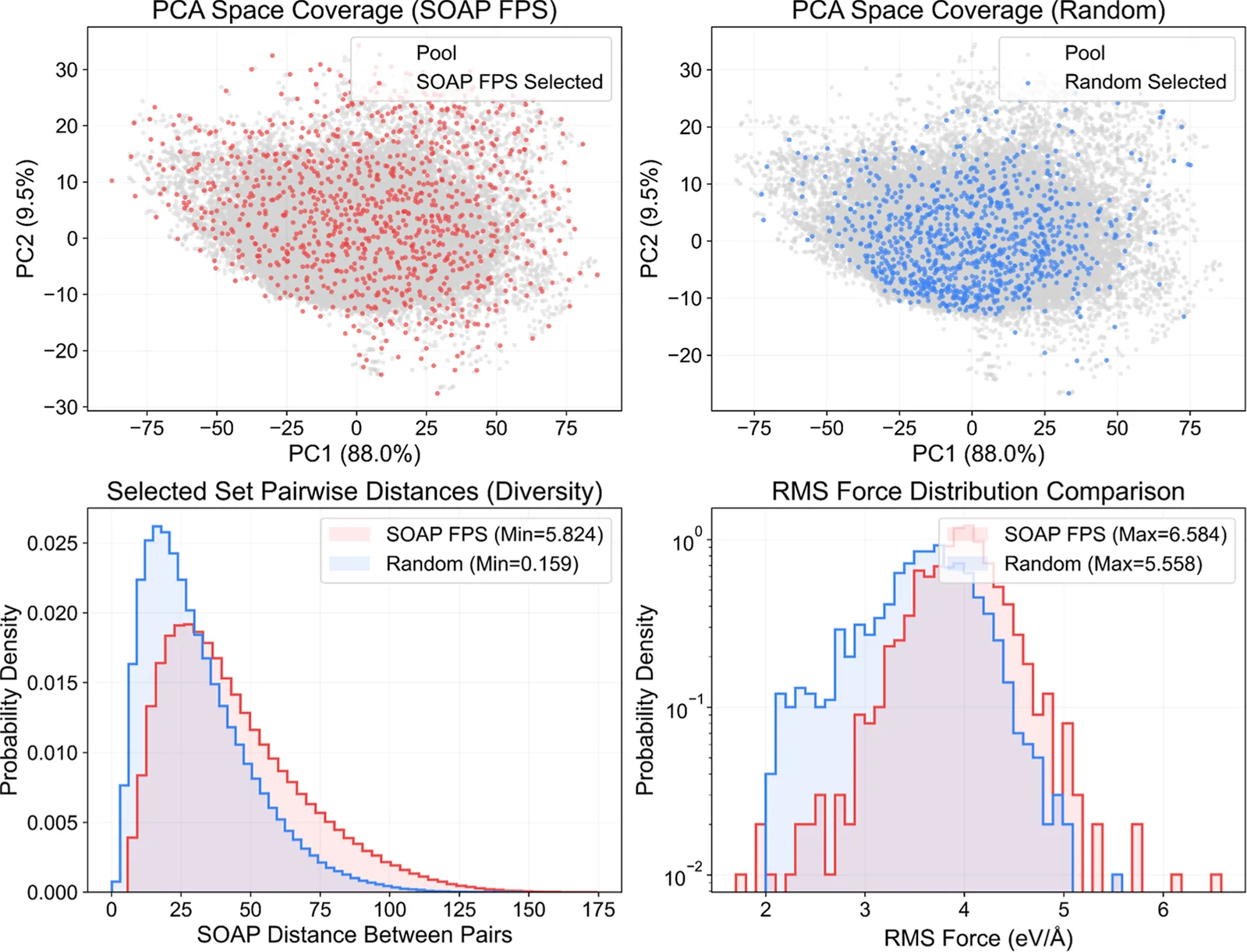
Quantitative analysis of structural diversity and configuration coverage for the active-learning-inspired selection compared to random sampling. (a,b) Coverage of the configurational space visualized through the first two principal components (PC1 and PC2) of the SOAP feature vectors for (a) snapshots selected via FPS and (b) randomly selected snapshots. The gray background represents the full pool of 40,000 snapshots. (c) Probability density of pairwise Euclidean distances between configurations in the SOAP feature space, demonstrating the significantly higher structural diversity (minimized redundancy) in the selected set. (d) Distribution of root-mean-square (RMS) atomic forces across the selected training sets.

Principal component analysis of the SOAP feature space clearly reveals that the FPS algorithm (Figure 3a) provides more uniform coverage of the configurational landscape compared to random sampling (Figure 3b), which tends to cluster configurations near the equilibrium center. This enhanced diversity is further confirmed by the distribution of pairwise distances in the feature space (Figure 3c); the selected snapshots exhibit substantially larger pairwise distances in the SOAP feature space, indicating that the approach eliminates redundant snapshots and identifies structural outliers. Furthermore, as shown in Figure 3d, the active-learning-inspired selection prioritizes configurations with higher root-mean-square forces (reaching 6.58 eV/Å), ensuring that the potential is exposed to high-energy, high-variance regions of the potential energy surface that are often underrepresented in random sampling.

The benefit of diversity-aware sampling can be seen from Figures 4 and S4 with quantitative results summarized in Table S3. While the differences between SOAP-selected and randomly sampled data sets remain relatively modest for the parent ZIF-8 framework, the SOAP-selected models exhibit improved performance on ZIF-8 in an unseen topology (i.e., dia), with the SOAP-selected 1000-configuration data set achieving a force MAE of 0.058 eV/Å compared with 0.077 eV/Å obtained using the randomly selected 1000-configuration data set. Consistent with this observation, the SOAP-selected model achieves predictive performance comparable to that obtained using substantially larger randomly sampled data sets, yielding a force MAE of 0.058 eV/Å for the unseen ZIF-8­(dia) topology, which is identical to the value obtained using 2000 randomly selected configurations.

**Figure 4:**
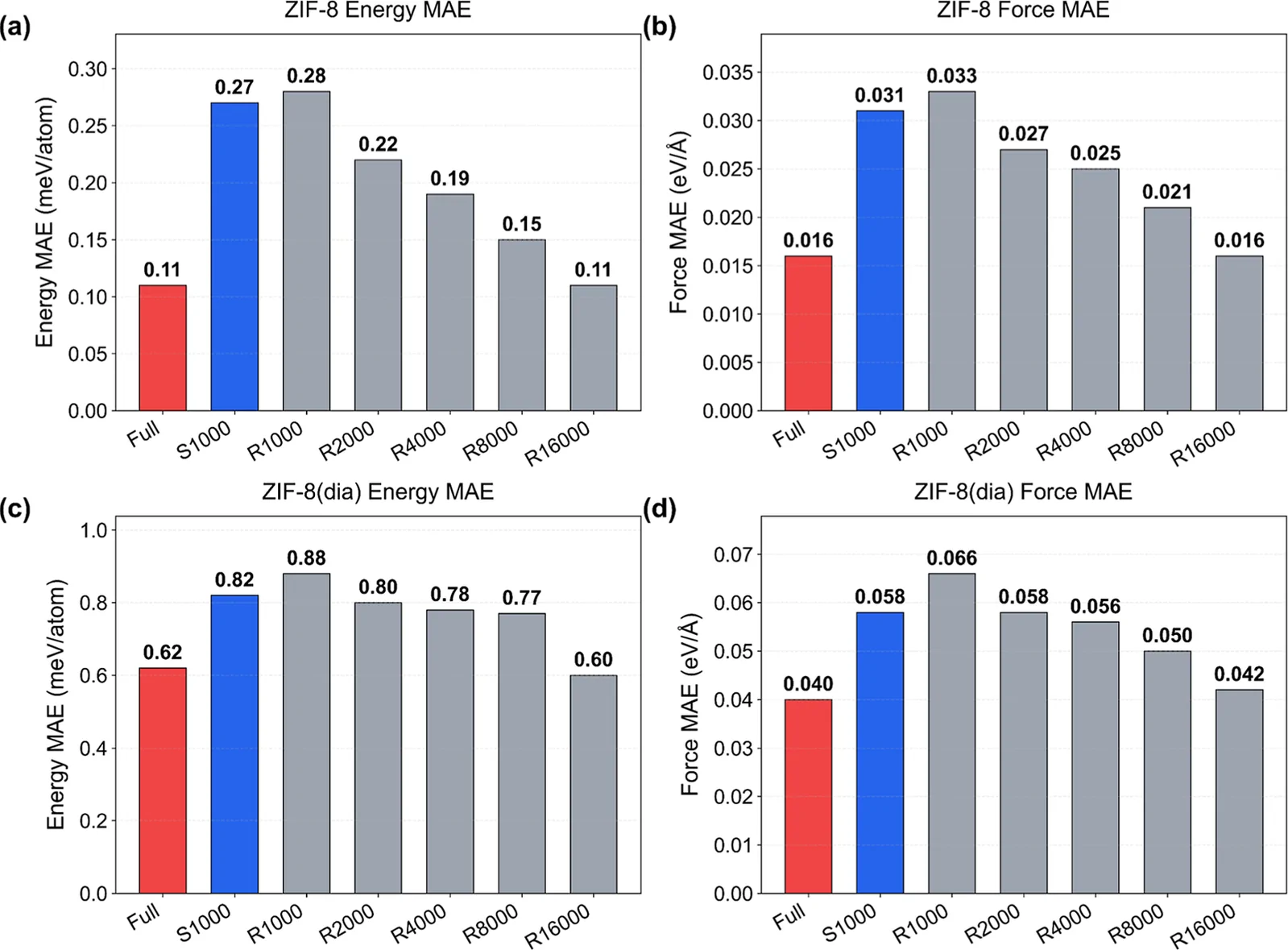
Evaluation of the SOAP-based structural selection strategy using ZIF-8 and ZIF-8­(dia) benchmark systems. Models trained on SOAP-selected configurations (S1000) are compared with randomly selected data sets of varying sizes (R1000, R2000, R4000, R8000, and R16000) and the full training data set. (a) Energy MAE for ZIF-8. (b) Force MAE for ZIF-8. (c) Energy MAE for ZIF-8­(dia). (d) Force MAE for ZIF-8­(dia).

These results suggest that diversity-aware sampling preferentially captures structurally informative configurations that improve transferability beyond the training topology. Note that the relatively modest difference between SOAP-selected and randomly sampled data sets observed herein likely arises from the broad configurational diversity already present in the original 40,000-snapshot data set, which is generated from AIMD simulations spanning temperatures from 300 to 2000 K. Additional tests using independent FPS initializations and different SOAP descriptor preprocessing yield similar learning behavior, indicating that the observed performance is not sensitive to a particular FPS initialization or descriptor scaling (see SI for details). Nonetheless, these results still demonstrate that the SOAP-based structural selection approach helps constructing representative training data sets and motivate its subsequent application to transferable MLP development.

### Foundational MLP for Zn-Coordinated ZIFs

Building upon the representative structural selection strategy established above, we next develop a transferable MLP across multiple Zn-coordinated ZIF frameworks. To ensure comprehensive coverage of functional groups and electronic environments, the training set is expanded beyond methyl-substituted linkers (i.e., ZIF-8) to include ZIF-7 (representing benzimidazole linkers with benzene resonance), ZIF-71 (incorporating chlorinated functional groups), and ZIF-90 (featuring aldehyde groups with oxygen atoms). The corresponding organic linkers employed in these frameworks are shown in Figure S1. To evaluate the transferability and robustness of the potential, another set comprising ZIF-8 in dia and kat topologies, as well as ZIF-11 and ZIF-72, is used. These frameworks serve as unseen frameworks for evaluating the transferability of the developed MLP.

The predictive performance in atomic force of the resulting transferable MLP is summarized in Figure 5, while the energy results can be seen in Figure S5. Their corresponding parity plots are provided in Figure S6. Similarly, to contextualize its performance, comparisons against other foundation models, including ORB, MACE, and CHGNet, on the same benchmark frameworks are also made. Note that comparisons with ReaxFF and UFF4MOF are not included in this analysis. As demonstrated from the ZIF-8 benchmark discussed above, both force fields exhibit substantially larger force prediction errors than the machine learning models considered herein. Across all frameworks investigated, our foundation MLP again consistently achieves the lowest force errors. For the frameworks represented in the training data set (ZIF-8, ZIF-90, ZIF-7, and ZIF-71), force MAEs range from 0.035–0.047 eV/Å, compared with 0.141–0.201 eV/Å for ORB, 0.221–0.383 eV/Å for MACE, and 0.337–0.404 eV/Å for CHGNet. Importantly, for unseen frameworks including ZIF-8­(dia), ZIF-8­(kat), ZIF-11, and ZIF-72, the established MLP maintains force MAEs below 0.10 eV/Å, whereas ORB produces errors of 0.141–0.196 eV/Å, MACE yields 0.214–0.373 eV/Å, and CHGNet yields 0.322–0.398 eV/Å. These results suggest that the developed foundation MLP captures transferable local atomic environments and interaction patterns, maintaining good predictive accuracy across frameworks with distinct linker chemistries, topologies, and lattice symmetries within the Zn-coordinated ZIF family.

**Figure 5:**
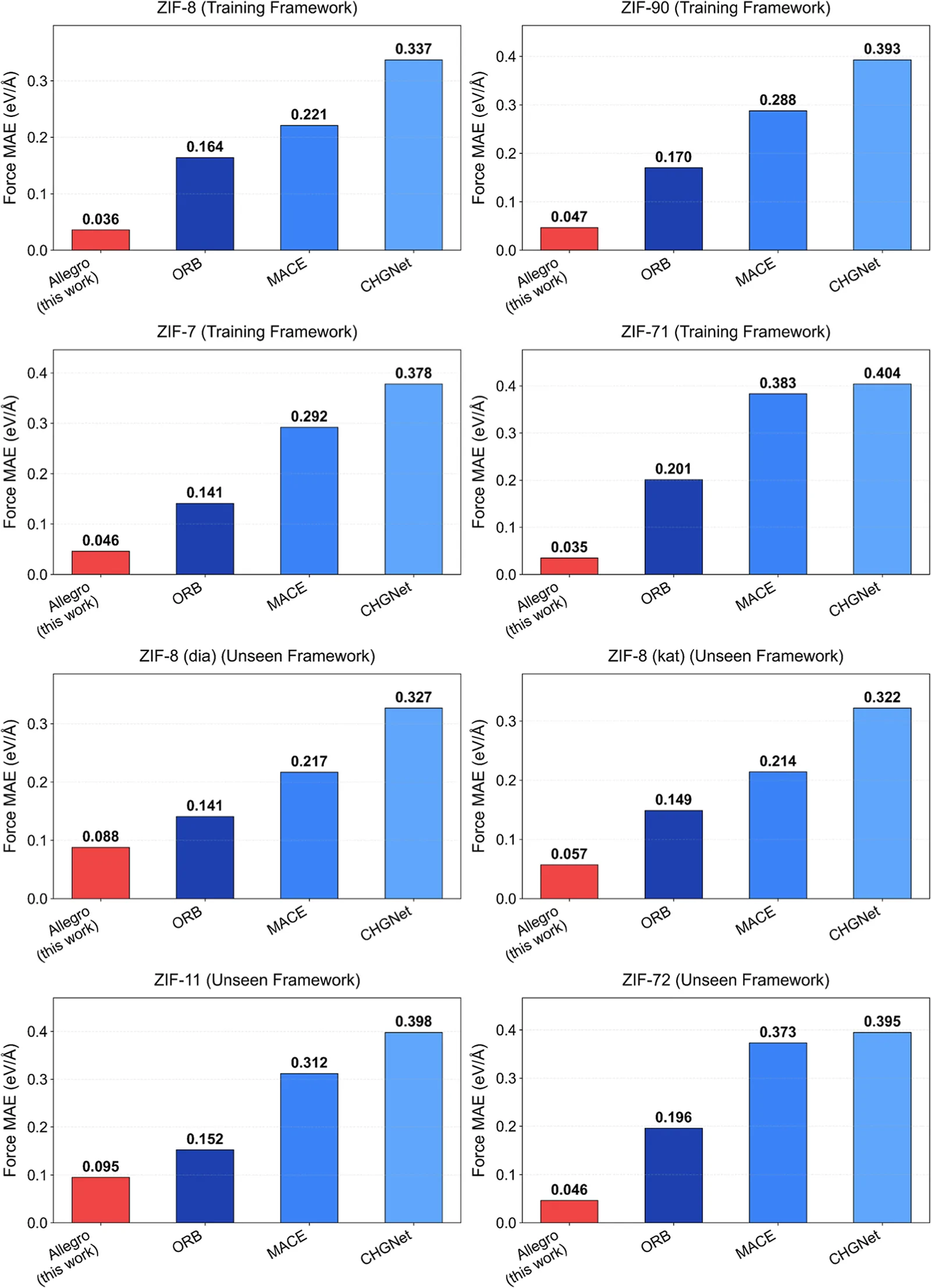
Comparison of force prediction accuracies for the transferable Allegro MLP developed in this study and state-of-the-art foundation models across Zn-coordinated ZIF frameworks. Force MAEs are evaluated on test data sets for the training frameworks (ZIF-8, ZIF-90, ZIF-7, and ZIF-71) and the unseen frameworks (ZIF-8­(dia), ZIF-8­(kat), ZIF-11, and ZIF-72). Numerical values are summarized in Table S4.

### Adaptability and EfficiencyThrough Transfer Learning

While the transferable MLP demonstrates encouraging performance across a broad range of Zn-coordinated ZIF frameworks, achieving framework-specific accuracy remains an important challenge. To this end, we investigate transfer learning as a hierarchical workflow that enables the above-discussed foundational MLP to be further efficiently specialized for targeted (but unseen) frameworks using only a limited number of additional configurations.^46,47^ This approach leverages the rich chemical vocabulary already acquired by a pretrained generalist model, allowing the neural network to be fine-tuned for specific structural secondary building units using only a fraction of the traditional data requirement.

To evaluate this strategy, we compare three distinct training regimes: (i) the above-discussed model utilizing 8000 snapshots per framework selected through our active-learning-inspired workflow (ZIF-8, ZIF-90, ZIF-7, and ZIF-71), (ii) a transfer learning model where the trained weights of foundational MLP are further fine-tuned with only 100 target-specific snapshots, and (iii) a model trained from scratch using the same 100 snapshots. Further learning-curve analysis using 10–500 target-specific configurations shows that the transfer-learning advantage persists over the investigated data range, with substantial improvement already achieved using very limited target-specific data (Figure S7). The benchmarking results for the unseen frameworks (i.e., for (i)) or the target structures (i.e., for (ii) and (iii)) are shown in Figure 6 with the tabulated values shown in Table S5. Notably, the transfer learning strategy is found highly effective for adapting the foundation MLP to alternative ZIF-8 topologies, reducing the force MAEs from 0.088 to 0.055 eV/Å for ZIF-8­(dia) and from 0.057 to 0.047 eV/Å for ZIF-8­(kat). Similar improvements are also observed for ZIF-11 and ZIF-72, where the force MAEs decrease from 0.095 to 0.063 eV/Å and from 0.046 to 0.041 eV/Å, respectively. For ZIF-72, the transfer-learned model also substantially outperforms the scratch model, which exhibits a force MAE of 0.210 eV/Å. The corresponding force and energy parity plots shown in Figure S8 further confirm the ability of the transfer-learned models to reproduce DFT reference data with higher fidelity. At this point, it is important to note that, while transfer learning substantially improves performance for the target frameworks, Figure S9 shows that, as would be anticipated, such fine-tuning may compromise the predictive accuracy on the original frameworks used for training the foundation MLP. This appears reasonable given that the fine-tuning process shifts the model toward the target structural domain, leading to a modest reduction in global generality. This behavior highlights the inherent trade-off between broad transferability and targeted specialization. Collectively, these results demonstrate that the Allegro framework can effectively transfer and refine the chemical knowledge learned from a generalist model to describe new structural environments with only a limited amount of additional data, offering an efficient route toward highly specialized and accurate MLPs.

**Figure 6:**
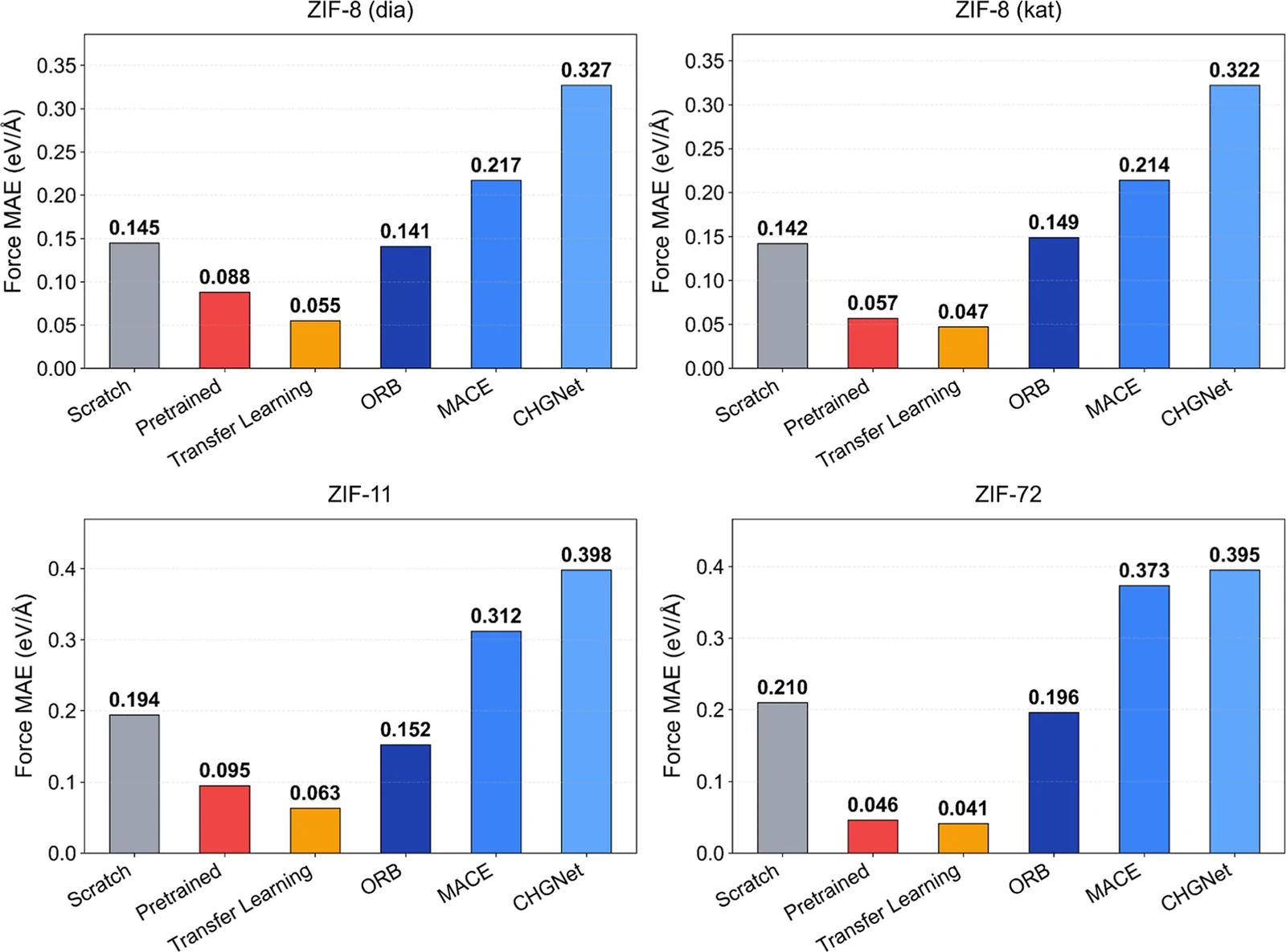
Comparative benchmarking of force prediction accuracy obtained using Scratch, Pretrained, and Transfer Learning strategies for unseen or targeted ZIF frameworks. Scratch models are trained using only 100 target-specific configurations, Pretrained models correspond to the transferable foundation MLP, and Transfer Learning models are obtained by fine-tuning the pretrained foundation model using the same 100 configurations. The evaluated frameworks include ZIF-8­(dia), ZIF-8­(kat), ZIF-11, and ZIF-72. Numerical values are summarized in Table S5.

### Case Study of Methane Permeation through Transient Pore-Window Opening

Having established the accuracy, transferability, and adaptability of the proposed potential, we next demonstrate its practical utility in large-scale MD simulations. That is, the applicability of the developed MLP is demonstrated through MD simulations of the methane permeation through transient pore-window opening in ZIF-8 as a representative case study. Note that, beyond the MLPs presented above for ZIFs, methane-containing ZIF-8 configurations are additionally incorporated into the training data set to also enable the description of host–guest interactions. The resulting potential maintains excellent predictive accuracy, yielding a small force MAE of 0.025 eV/Å as illustrated in Figures S10 and S11. Such DFT-level accuracy should provide a reliable foundation to investigate methane-in-ZIF-8 systems. Herein, MLP-powered MD simulations are conducted through the implementation in the open-source LAMMPS package. Previous experimental adsorption isotherms as introduced above indicate that methane adsorption in ZIF-8 exhibits more pronounced adsorption-step behavior at lower temperatures,^24^ and therefore simulations are conducted at 200 K. To benchmark the framework dynamics of methane-loaded ZIF-8 against a conventional force-field description, additional flexible-framework MD simulations were performed using UFF4MOF at 200 K under the same methane loading and simulation-cell conditions. The UFF4MOF simulation reproduces the dominant near-equilibrium linker fluctuations but exhibits a noticeably narrower swing-angle distribution than the MLP. As shown in Figure S12, the MLP samples broader and larger-amplitude linker fluctuations relevant to transient pore-window opening.

As illustrated in Figure 7, the simulation cell consists of a periodic ZIF-8 sodalite framework loaded with seven methane molecules acting as guest probes. A defining characteristic of ZIF-8 is its intrinsic lattice flexibility, where the nominal pore window of approximately 3.4 Å must expand to accommodate the kinetic diameter of methane (3.8 Å). The developed potential successfully captures a rare methane permeation event through a transiently opened pore window, which remains computationally challenging to sample directly using conventional AIMD. To visualize the permeation pathway, Figure 7e presents a focused view of a representative methane transport event through a six-membered-ring pore window. Initially, the methane molecule resides near the exterior of the pore aperture. As the methane molecule approaches the window, local linker reorientation enlarges the pore aperture and facilitates guest permeation. The methane molecule subsequently traverses the window and enters the adjacent sodalite cage. To quantify methane transport beyond this representative event, multiple independent trajectories were further analyzed for cage-to-cage hopping. At 200 K, approximately 51 ns·CH4 of aggregate exposure was sampled per observed crossing, whereas at 400 K, three crossings were observed over 5.1 ns·CH_4_, corresponding to an observed hopping rate of approximately 0.59 crossings·CH_4_^–1^·ns^–1^. Although the higher hopping frequency at 400 K indicates a strong temperature dependence of methane transport, the limited number of long-range transitions precludes extraction of a statistically converged self-diffusion coefficient.

**Figure 7:**
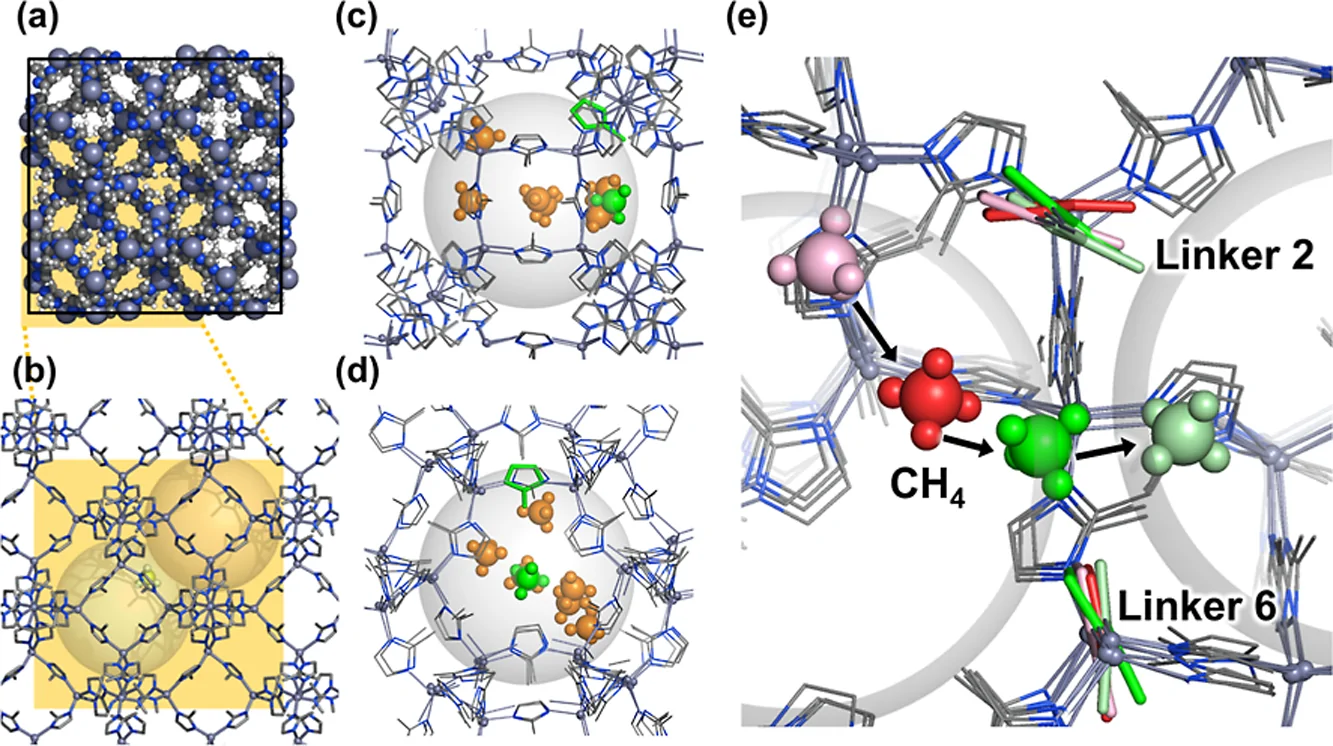
Structural visualization of methane permeation through the ZIF-8 pore window during a gate-opening event. (a) Periodic simulation cell used for the methane-loaded ZIF-8 simulation. (b) Enlarged view of the pore network highlighted in panel (a). (c,d) Representative configurations showing the methane molecule highlighted in green approaching the pore window from different viewing directions, while the remaining methane molecules are shown in orange. The six-membered-ring window involved in the permeation event is highlighted in green. (e) Sequential snapshots illustrating methane transport through the pore window before (red), during (green), and after (light green) the gate-opening event. The colored planes correspondingly represent the orientations of the imidazolate linkers defining the pore aperture during permeation. Carbon, nitrogen, hydrogen, and Zn atoms are shown in gray, blue, white, and purple, respectively.

This structural evolution is directly corroborated by the dynamical analysis presented in Figure 8a that shows the methane-to-window distance profile as a function of simulation time. The abrupt transition in the methane–window distance profile provides quantitative evidence of successful guest transport through the framework aperture. To elucidate the underlying mechanism, the rotational dynamics of three imidazolate linkers surrounding the pore window (Linkers 2, 4, and 6; their locations are illustrated in Figure S13) are analyzed with results shown Figure 8b. These linkers are selected because their methyl groups are oriented toward the pore window and thus play a direct role in the steric environment experienced by the permeating methane molecule. Corroboratively, a pronounced rotation of Linker 2 is observed, reaching a peak plane angle of approximately 87°. However, analysis of multiple trajectories does not provide sufficient statistics to establish a reproducible temporal ordering between linker rotation and methane passage. We therefore interpret the observed linker reorientation and pore-window opening as framework fluctuations associated with methane permeation rather than assigning a causal sequence to these motions. Relative orientations of Linkers 2 and 4 can also be visually seen from Figure 7e. Collectively, these results show that our MLP effectively bridges the gap between accurate electronic structure calculations and large-scale sampling, providing a physically realistic tool for the atomic-level characterization and rational design of next-generation separation materials.

**Figure 8:**
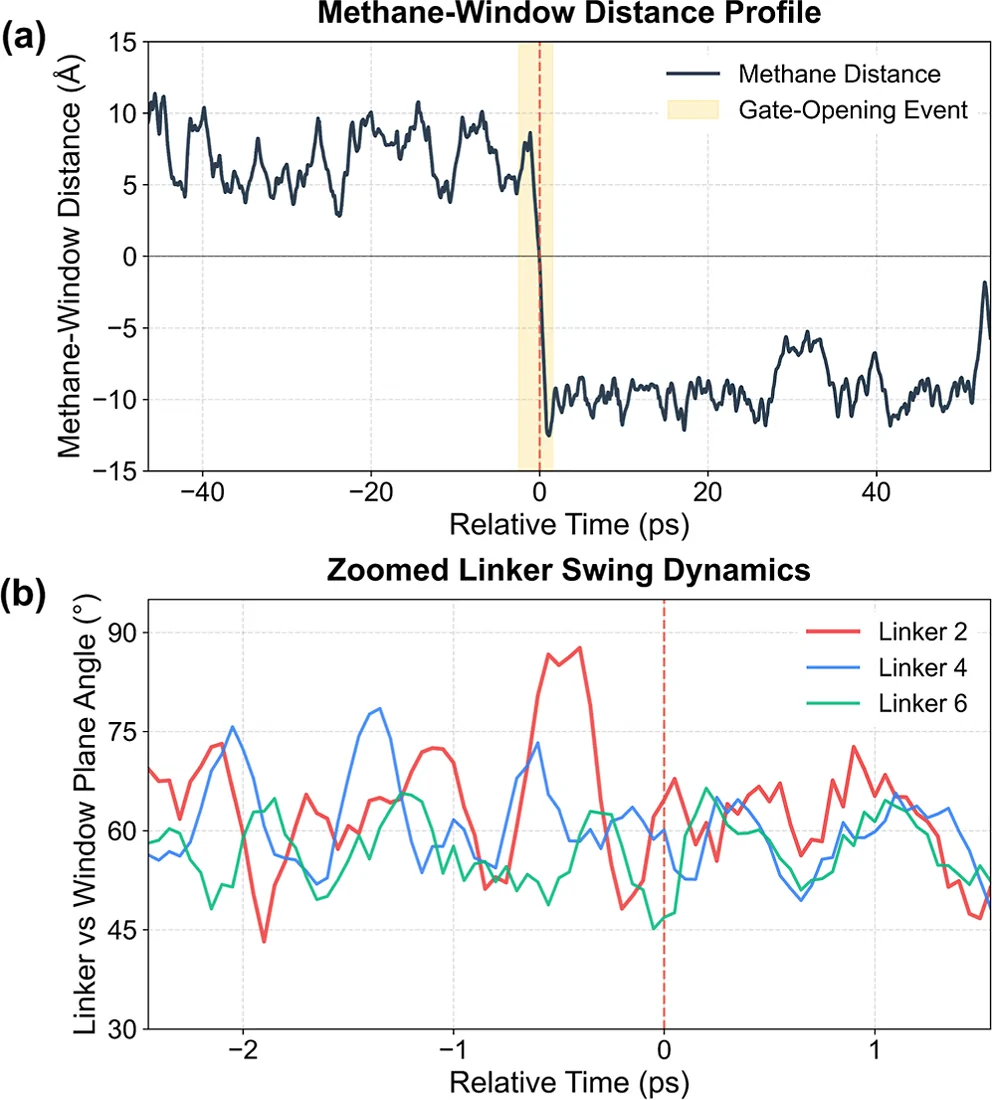
Dynamical analysis of a representative methane cage-to-cage permeation event in ZIF-8. (a) The distance between the methane molecule and the center of the target pore window over a 100 ps trajectory, with the highlighted region marking the successful insertion event. (b) A zoomed-in view of the angular dynamics of the three imidazolate linkers forming the window. In this figure, the *x*-axis represents the relative time with respect to the observed permeation event.

## Conclusions

In this study, a transferable MLP is developed for the Zn-coordinated ZIF family using the equivariant Allegro architecture. Systematic benchmarking against first-principles calculations demonstrates that the developed MLP accurately reproduces the potential energy surfaces of diverse ZIFs with DFT-level fidelity as well as consistently outperforming classical force fields, reactive force fields, and several recently developed MLPs. To construct accurate MLPs, we establish a hierarchical workflow that includes SOAP-based structural selection, extensive framework training, and a transfer learning strategy. The SOAP-based sampling strategy enables the construction of representative training data sets by identifying structurally informative configurations. The transferable foundation model successfully captures diverse Zn-coordinated ZIFs, including several unseen framework topologies and chemical environments not represented in the training data set. Furthermore, transfer learning enables efficient specialization toward targeted but unseen systems using as few as 100 additional configurations while substantially reducing the computational cost associated with generating large-scale reference data sets. The practical utility of the developed potential is further demonstrated through MLP-powered MD simulations that capture methane-induced gate-opening in ZIF-8 over spatial and temporal scales inaccessible to conventional AIMD. Beyond MD, the developed MLP could also be integrated with GCMC-based adsorption sampling, for example, by describing framework–guest interactions with the MLP and guest–guest interactions with a classical force field, while incorporating framework flexibility through intermittent MLP-driven MD relaxation. Along with this study, the trained MLPs and associated deployment files are provided in the SI, facilitating future simulations of Zn-coordinated ZIF systems.

## Supplementary Material





## Data Availability

**Data and Software Availability** All detailed computational settings, training protocols, benchmarking results, and supporting analyses are provided in the Supporting Information. The MLPs developed in this work are also included as the Supporting Information in PyTorch (.pth) format and can be directly implemented through the NequIP/Allegro framework for MD simulations in LAMMPS. The DFT reference data sets and training configuration files are publicly available through Zenodo (10.5281/zenodo.22314232).

## Notes

Computational details including DFT data set generation protocols, SOAP-based structural selection analysis, Allegro MLP training protocol, and performance benchmarks. Additional figures and tables, including parity plots, benchmarking results against foundation models and conventional force fields, transfer learning performance evaluations, methane-induced gate-opening simulations (PDF) (PDF)
MLP for ZIF-8 (ZIF8.nequip.pth) (pth). Transferable MLP for Zn-coordinated ZIFs (ZIFall.nequip.pth) (pth). Transfer-learned MLP obtained by further fine-tuning the transferable foundation model on the target framework data sets (ZIF-8­(dia), ZIF-8­(kat), ZIF-11, and ZIF-72) (ZIFall_TL.nequip.pth) (pth). MLP including methane–framework interactions for gate-opening simulations (ZIF8_CH4.nequip.pth) (pth) (ZIP)
