# Supplementary material for: Efficient and Transferable Machine Learning Potentials for Zn-Coordinated Zeolitic Imidazolate Frameworks Through Structural Diversity Sampling and Transfer Learning
Source: J Chem Theory Comput. 2026 Sep 30;22(19):10391–402. doi: 10.1021/acs.jctc.6c01271 (PMC13671164; doi:10.1021/acs.jctc.6c01271)
Supplement: Supplementary file 1 [file ct6c01271_si_002.pdf]

Supporting Information

for

Efficient and Transferable Machine Learning Potentials for Zn-Coordinated Zeolitic Imidazolate Frameworks through Structural Diversity Sampling and Transfer Learning

Shang-Wei Lin[a]*, Yen-Yung Wu[a], Li-Chiang Lin[a]*

[a]*Department of Chemical Engineering, National Taiwan University, Taipei, 10617, Taiwan*

Email: qwert75811@gmail.com (S.-W. Lin) ; lclin@ntu.edu.tw (L.-C. Lin)

Table of Contents

1. Computational details 3
2. Additional Figures referred in the main text 9
3. Additional Tables referred in the main text 23
4. References 28

1. Computational details

1.1 DFT Reference Data Generation

As noted in the main text, reference configurations used for the training and testing of MLPs are generated using ab initio molecular dynamics (AIMD) simulations performed with the CP2K package (version 2025.2).[1] Electronic structure calculations are carried out using the Quickstep module with the Perdew–Burke–Ernzerhof (PBE) exchange-correlation functional in conjunction with the DFT-D3(BJ) dispersion correction. The DZVP-MOLOPT-SR-GTH basis sets and Goedecker–Teter–Hutter (GTH) pseudopotentials are employed for all elements.[2] All calculations are performed under periodic boundary conditions using the GPW formalism with a plane-wave cutoff of 600 Ry and a relative cutoff of 40 Ry. Self-consistent field calculations employ the orbital transformation (OT) method with a convergence criterion of 1.0×10^{-5} Ha. All DFT calculations and AIMD simulations are performed on the Taiwania 3 and Frontier 1 high-performance computing systems operated by the National Center for High-performance Computing (NCHC), Taiwan, using 56 CPU cores per simulation. Taiwania 3 employs dual-socket Intel Xeon Platinum 8280L processors (56 cores per node), whereas Frontier 1 employs dual-socket Intel Xeon Platinum 8480+ processors (112 cores per node).

Configurational sampling is performed through AIMD simulations in the canonical (NVT) ensemble using a canonical sampling through velocity rescaling (CSVR) thermostat with a time constant of 1000 fs. A timestep of 0.5 fs is employed for all simulations. For the ZIF-8 framework, AIMD trajectories are generated from 300 K to 2000 K at intervals of 100 K. For ZIF-7, ZIF-71, ZIF-90, ZIF-8(dia), ZIF-8(kat), ZIF-11, and ZIF-72, AIMD simulations are performed at 300, 500, 800, 1000, 1300, 1500, 1800, and 2000 K. This temperature protocol enables efficient sampling of framework flexibility, linker rotations, thermal distortions, and high-energy configurations across diverse ZIF topologies and chemical environments.

The AIMD simulations generate atomic coordinates, forces, cell vectors, and total energies at every simulation step. These outputs are subsequently parsed and combined into the extended XYZ (extxyz) format using in-house post-processing scripts. For the ZIF-8 dataset, 50,000 configurations are sampled from AIMD trajectories and subsequently divided into training, validation, and test

subsets using an 8:1:1 ratio, yielding 40,000, 5,000, and 5,000 configurations, respectively. For the transferable foundation MLP development, 8,000 representative configurations are selected from each framework (ZIF-8, ZIF-90, ZIF-7, and ZIF-71) using the SOAP-based farthest point sampling (FPS) strategy and subsequently used for training. Validation and testing datasets are subsequently constructed by randomly selecting 1,000 configurations each from the remaining trajectory pool.

1.2 SOAP-Based Structural Selection

To evaluate the effectiveness of diversity-aware sampling, the SOAP-based selection procedure is first benchmarked using the 40,000-configuration ZIF-8 training dataset generated from the DFT reference database as mentioned above.[3] The same protocol is subsequently applied to all frameworks used for the transferable MLP development. SOAP descriptors are calculated using a radial cutoff of 6.0 Å, with the expansion of the local atomic density truncated at nmax = 4 and lmax = 4. Periodic boundary conditions are included during descriptor generation, and Zn, C, N, H, O, and Cl atoms are considered in the descriptor construction. To represent each structure as a single feature vector, the inner averaging scheme is employed, resulting in a global descriptor suitable for structural similarity analysis.

The resulting SOAP descriptors are used as inputs for the FPS algorithm. The first configuration is selected randomly from the training pool. At each subsequent iteration, the Euclidean distance between all remaining structures and the previously selected set is evaluated in SOAP feature space. The configuration exhibiting the largest minimum distance to the selected subset is then added to the training set. This process is repeated until the desired number of representative structures is obtained. Following this procedure, representative datasets containing 1,000 (S1000) and 2,000 (S2000) configurations are generated.

For comparison, randomly sampled datasets are generated from the same 40,000-frame training pool using an identical random seed. Random subsets containing 1,000 (R1000), 2,000 (R2000), 4,000 (R4000), 8,000 (R8000), and 16,000 (R16000) configurations are constructed. All SOAP-selected and randomly sampled datasets are subsequently used to train independent Allegro models for evaluating

the impact of structural diversity on predictive accuracy and data efficiency. To assess the robustness of the SOAP-FPS selection, the FPS procedure is additionally repeated using three independent initializations. Although the resulting 1,000-configuration subsets exhibit only approximately 26% pairwise overlap, the corresponding force MAEs vary by only 0.5–6% of the mean, indicating that the model performance is not strongly dependent on the initial FPS seed. In addition, repeating the selection using standardized SOAP descriptors substantially changes the selected configurations, with only 7.8–8.9% overlap with the original selections, while yielding similar learning behavior. These results indicate that the observed performance of SOAP-FPS does not depend strongly on a particular FPS initialization or descriptor preprocessing. The temperature distribution of the SOAP-FPS-selected configurations is further analyzed in Figure SX. Configurations spanning the entire temperature range are retained, while higher-temperature trajectories contribute more strongly to the selected dataset, consistent with the preferential selection of thermally distorted configurations.

1.3 Allegro Training Protocol

MLPs are developed using the Allegro framework (version 0.7.0) implemented within the NequIP package.[4] The training environment utilizes Python 3.10, CUDA 12.6, PyTorch Lightning, and the NequIP/Allegro software stack on the Nano 5 supercomputing system operated by NCHC. The model employs a radial cutoff of 6.5 Å and includes Zn, C, N, H, O, and Cl as atomic species. Local atomic environments are represented using an eight-function Bessel radial basis expansion combined with polynomial cutoff functions. The final architecture consists of two equivariant interaction layers with a maximum angular momentum of (l_{max} = 1), 128 scalar features, and 32 tensor features. Parity symmetry is explicitly preserved, and SiLU activation functions are employed throughout all multilayer perceptron blocks.

Model training is performed using PyTorch Lightning on a single NVIDIA H100 GPU. The training, validation, and test datasets are loaded through the ASEDataModule implemented in NequIP. Data shuffling is enabled during training, and eight CPU cores are allocated for data loading. A batch size of one structure is employed during training, with gradient accumulation over two optimization

steps to improve memory efficiency. Model parameters are optimized using the Adam optimizer with an initial learning rate of 0.001. A ReduceLROnPlateau scheduler is employed to dynamically reduce the learning rate by a factor of 0.5 whenever the validation metric fails to improve for ten consecutive epochs. Training is allowed to proceed for up to 1000 epochs, while early stopping is triggered when the monitored validation metric fails to improve for 30 consecutive epochs. Exponential moving average (EMA) weights with a decay factor of 0.999 are additionally maintained throughout training to improve model stability.

The loss function is defined as a weighted sum of per-atom energy and force errors, with equal weighting coefficients assigned to both contributions. Model performance is monitored using the weighted sum of energy and force errors. TensorFloat-32 (TF32) acceleration is enabled during training to improve computational efficiency on NVIDIA GPU architectures. Per-type energy shifts and force scales are initialized using statistics computed from the training dataset and remain fixed throughout optimization. Training and validation progress are monitored through both Weights & Biases and CSV-based logging, and the final model parameters correspond to the checkpoint exhibiting the lowest validation loss. For evaluation of energy prediction errors, a single constant energy offset is determined separately for each model from the mean signed difference between the predicted and DFT reference energies on the validation set. This offset is then fixed and applied unchanged to the corresponding test set. No test-set reference energies are used in determining the energy correction. Because a constant energy shift does not affect atomic forces, the reported force errors require no corresponding correction.

1.4 Performance Benchmark

To evaluate the practical deployment efficiency of the developed potential, inference benchmarks were performed on a local workstation equipped with an Intel Core i9-14900K processor and an NVIDIA GeForce RTX 5080 GPU. The benchmark results reported in **Table S2** include force prediction accuracy, MD throughput, and peak GPU memory consumption for the developed Allegro potential and the foundation models ORB v3, MACE-medium, and CHGNet using the same 276-atom

ZIF-8 benchmark system. Among these models, ORB v3, MACE-medium, and CHGNet achieve MD throughputs of 1.395, 1.134, and 0.609 ns/day, respectively, compared with 4.679 ns/day for the developed Allegro potential. In addition, we specifically benchmarked MACE-MP-MOF0, a MACE model fine-tuned for MOF systems, to provide a more relevant comparison with a MOF-specialized foundation model. MACE-MP-MOF0 achieves a force MAE of 0.092 eV/Å, an MD throughput of 1.094 ns/day, and a peak GPU memory consumption of 1324 MB. Under the same benchmark conditions, the developed Allegro potential achieves a substantially lower force MAE of 0.016 eV/Å, approximately 4.3-fold higher MD throughput (4.679 vs 1.094 ns/day), and requires only 29% of the GPU memory (384 vs 1324 MB). These results demonstrate that the system-specific Allegro potential provides substantial advantages in both predictive accuracy and computational efficiency relative to the MOF-specialized foundation model for the ZIF-8 system considered here.

Note that, for consistency and reproducibility, the Allegro potential is evaluated using the standard TorchScript deployment format without architecture-specific optimizations. However, recent developments in the NequIP/Allegro framework have demonstrated that additional deployment strategies, including Ahead-of-Time Inductor (AOTI) compilation and custom tensor-product kernels, can substantially improve inference throughput and memory efficiency on modern GPU architectures, particularly NVIDIA H100 accelerators. In MD benchmarks, these optimizations have been reported to accelerate Allegro inference by up to an order of magnitude relative to conventional TorchScript deployment.[5] Therefore, the performance metrics reported in **Table S2** should be regarded as conservative estimates of the achievable inference efficiency of the Allegro framework.

1.5 Validation of Local Structure against AIMD

To further evaluate the reliability of the developed MLP for finite-temperature MD simulations, the local structure of ZIF-8 obtained from the MLP trajectory was compared with that sampled by the CP2K AIMD reference trajectory. **Figure S2** compares the Zn–N and Zn–Zn radial distribution functions (RDFs) and the integrated Zn–N coordination numbers. The first Zn–N RDF peak occurs at 1.99 Å for both AIMD and MLP, while the first Zn–Zn peaks are located at 5.97 and 5.95 Å,

respectively. The difference in their broadness may be attributed to the different thermal control approaches and the notably shorter timescale accessible to the AIMD simulations. Integration of the Zn–N RDF also yields a coordination number of 4.00 for both simulations, confirming preservation of the tetrahedral ZnN_4 coordination environment. These results show that the MLP accurately reproduces the characteristic local coordination geometry sampled by the DFT reference trajectories.

1.6 Framework Dynamics of Methane-Loaded ZIF-8

To further benchmark the framework dynamics of methane-loaded ZIF-8, additional flexible-framework MD simulations were performed using UFF4MOF at 200 K and 1 bar under the same methane loading and simulation-cell conditions as the MLP simulations. As shown in **Figure S12**, both models exhibit linker swing-angle distributions centered near the equilibrium orientation, indicating qualitatively similar dominant linker fluctuations. However, UFF4MOF produces a noticeably narrower distribution, whereas the MLP samples a broader range of linker orientations with more pronounced large-amplitude excursions. These results indicate that UFF4MOF captures the dominant near-equilibrium linker motion but provides a more restricted description of the larger framework fluctuations relevant to transient pore-window opening.

2. Additional Figures referred in the main text

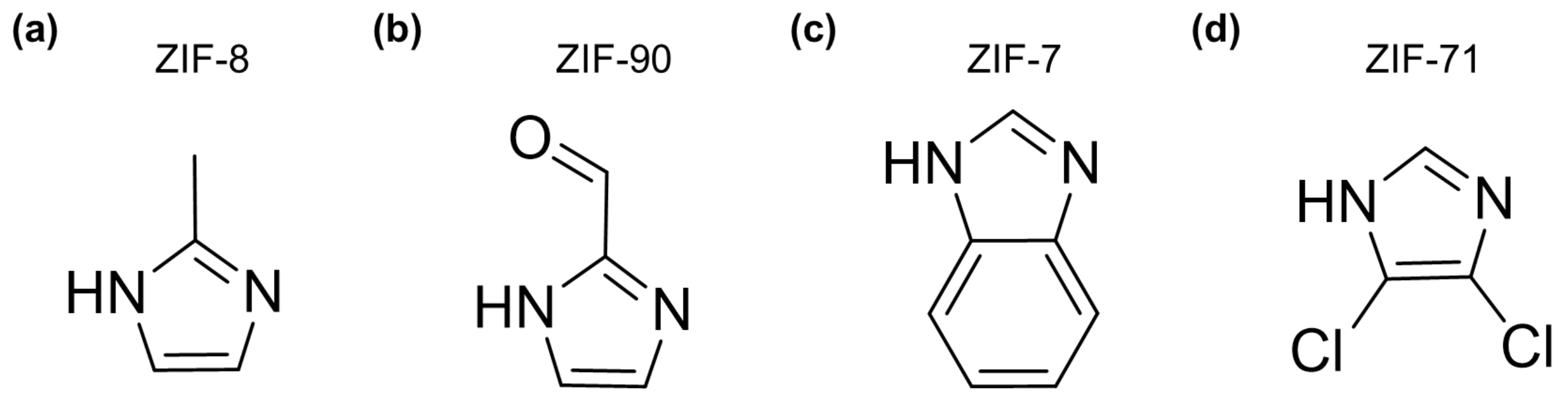

Figure S1. Organic linkers employed in (a) ZIF-8, (b) ZIF-90, (c) ZIF-7, and (d) ZIF-71 investigated in this study. The corresponding linkers are 2-methylimidazole, 1H-imidazole-2-carbaldehyde, benzimidazole, and 4,5-dichloroimidazole, respectively.

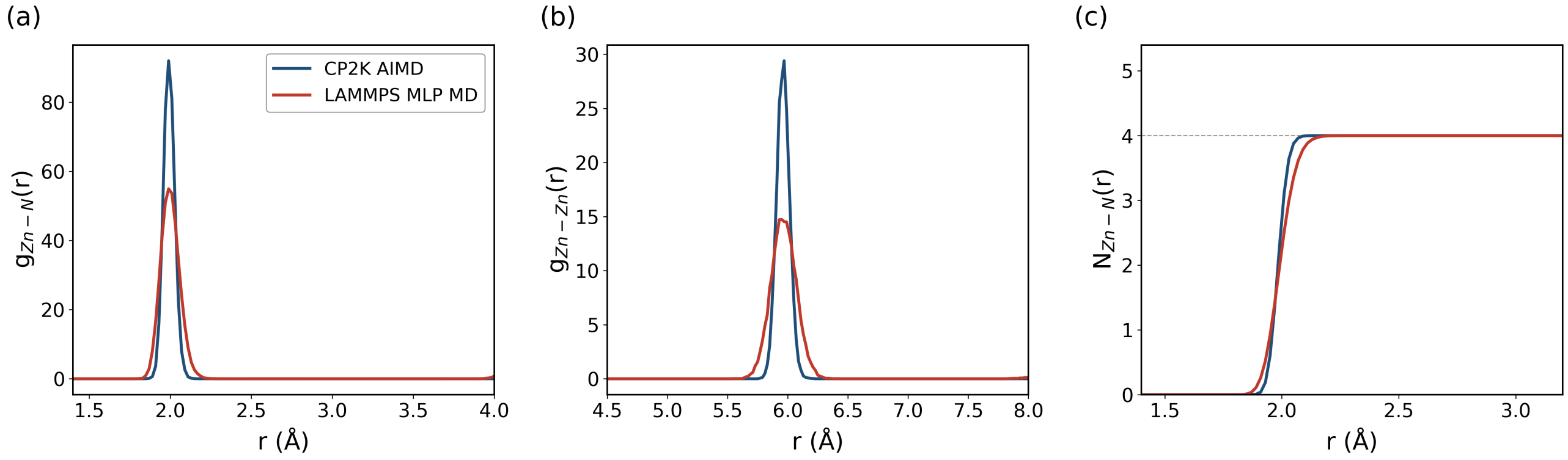

Figure S2. Comparison of the local structure of ZIF-8 obtained from CP2K AIMD and LAMMPS MLP simulations. (a) Zn–N radial distribution function, (b) Zn–Zn radial distribution function, and (c) integrated Zn–N coordination number. The MLP reproduces the characteristic Zn–N and Zn–Zn distances and maintains the four-fold Zn coordination observed in the AIMD reference trajectory.

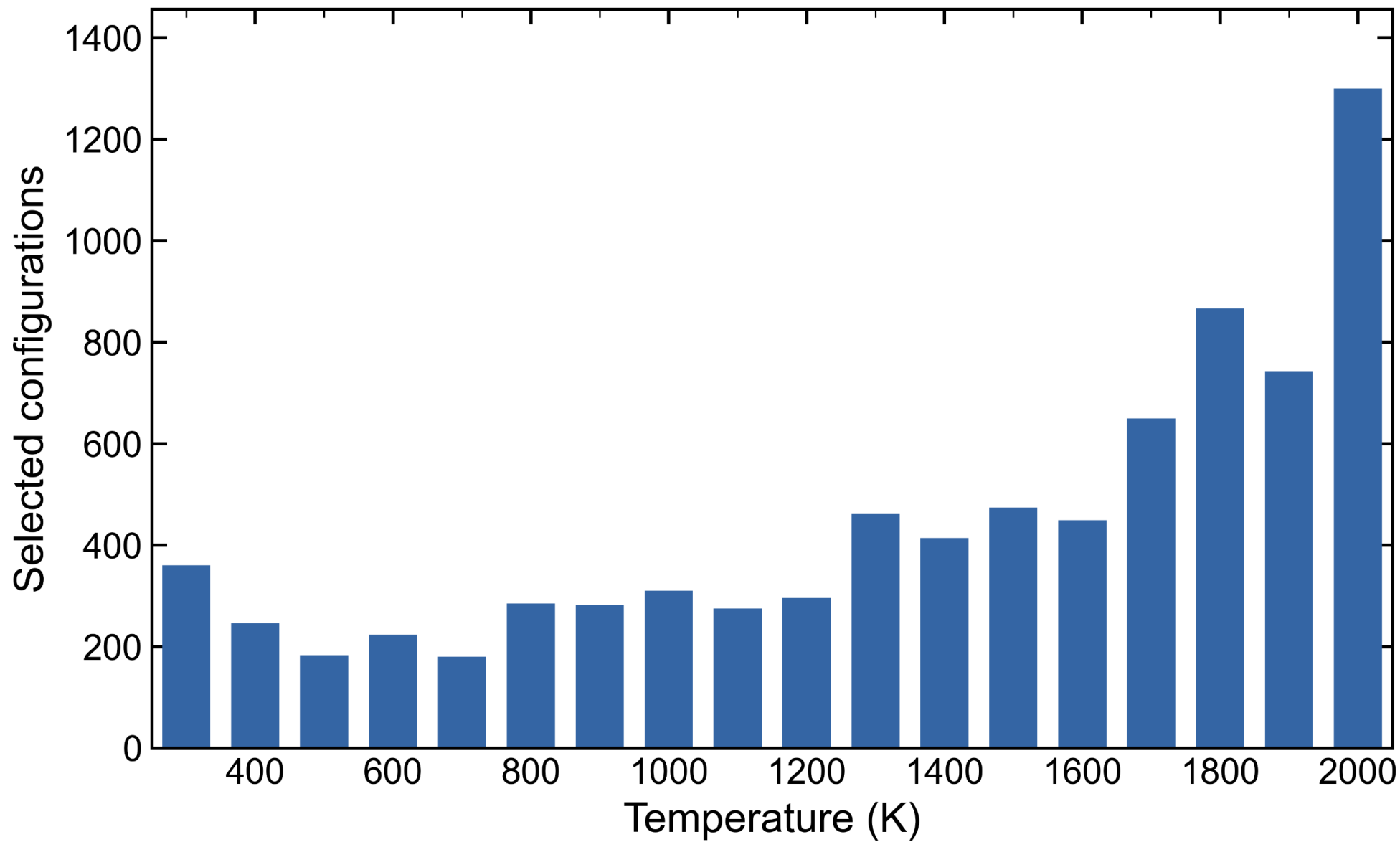

Figure S3. Temperature distribution of configurations selected by SOAP-based farthest point sampling (FPS) from the ZIF-8 AIMD dataset.

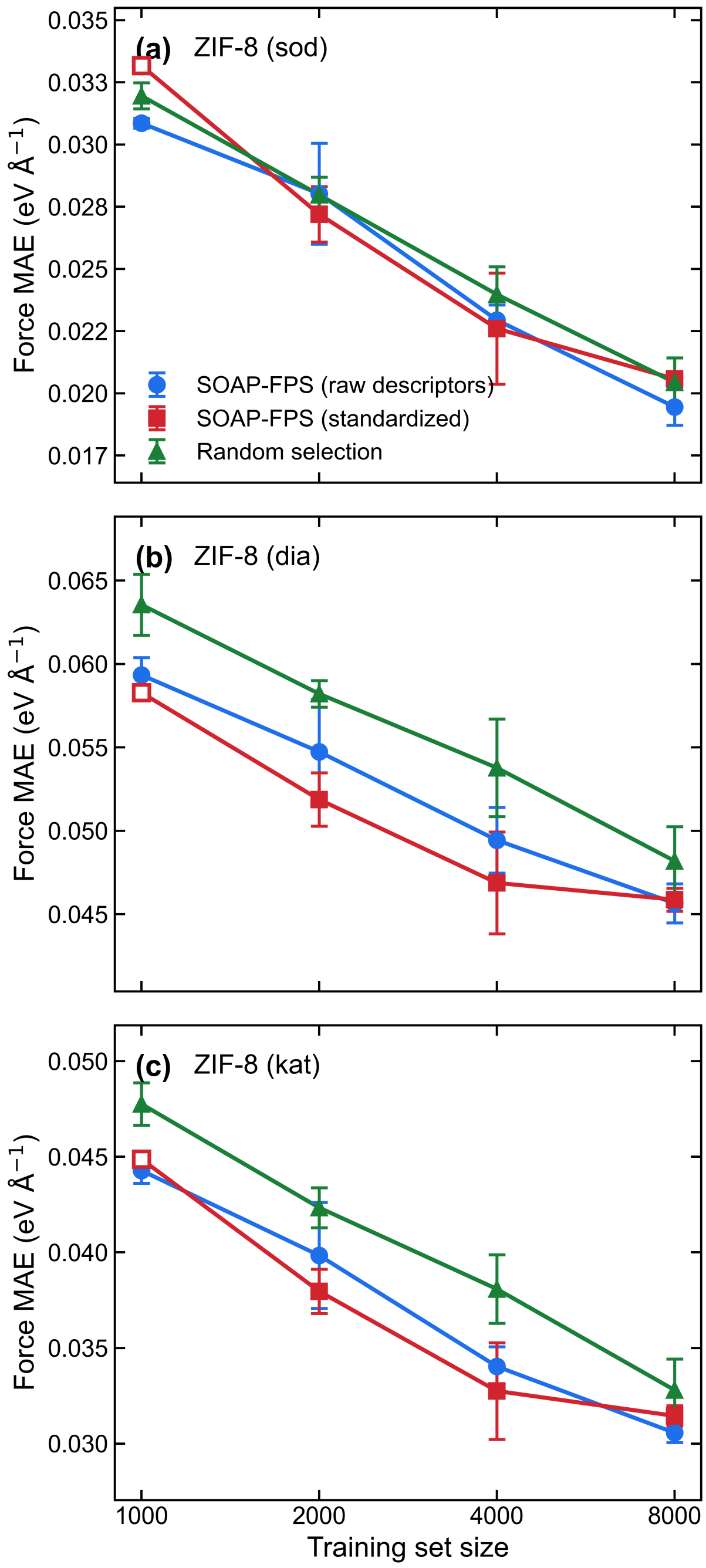

Figure S4. Robustness of SOAP-based farthest point sampling (FPS) with respect to the FPS initialization. Force MAEs obtained for (a) ZIF-8, (b) ZIF-8(dia), and (c) ZIF-8(kat) as a function of the number of training configurations. SOAP-FPS results are reported as the mean and standard deviation over three independent FPS initializations. The small variation among independent selections indicates that the observed learning behavior is not sensitive to the initial FPS seed.

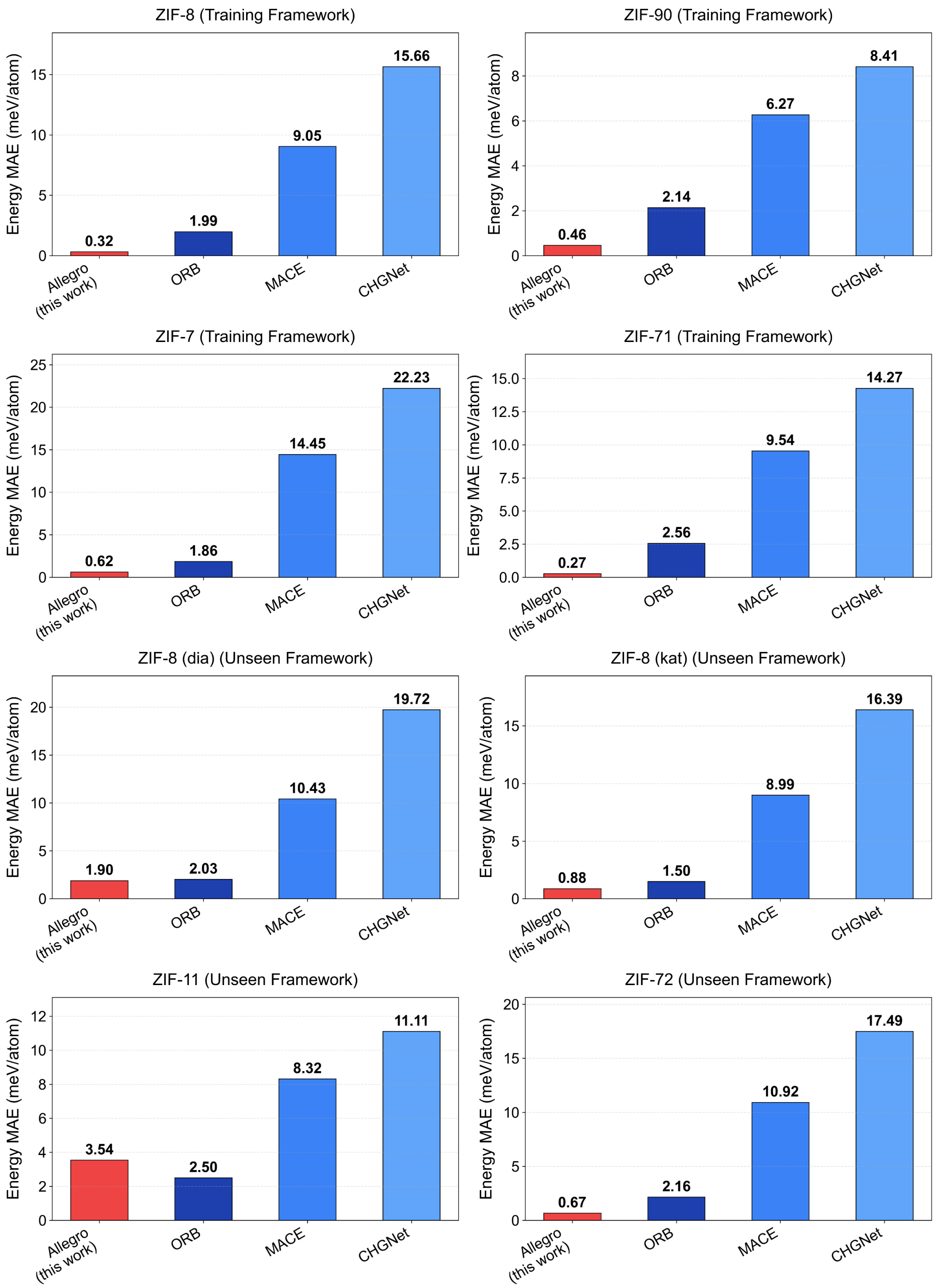

Figure S5. Comparison of energy prediction accuracy among Allegro (this work), ORB, MACE, and CHGNet for Zn-coordinated ZIF frameworks. Energy MAEs are shown for the training frameworks (ZIF-8, ZIF-90, ZIF-7, and ZIF-71) and unseen frameworks (ZIF-8(dia), ZIF-8(kat), ZIF-11, and ZIF-72).

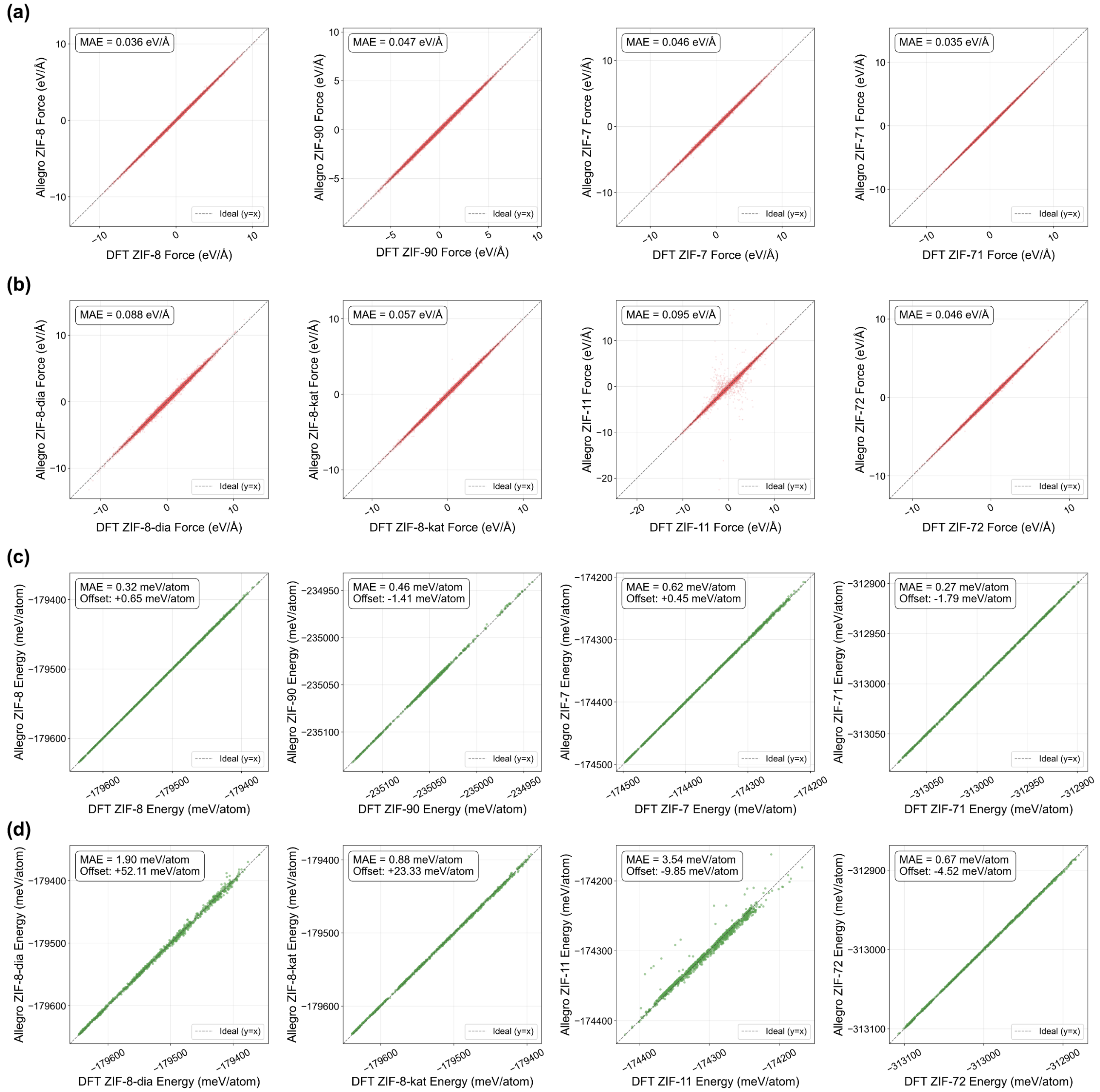

Figure S6. Force parity plots for the transferable Zn-coordinated ZIF MLP showing (a) the training frameworks and (b) the unseen frameworks. Atomic energy parity plots for the transferable Zn-coordinated ZIF MLP showing (c) the training frameworks and (d) the unseen frameworks.

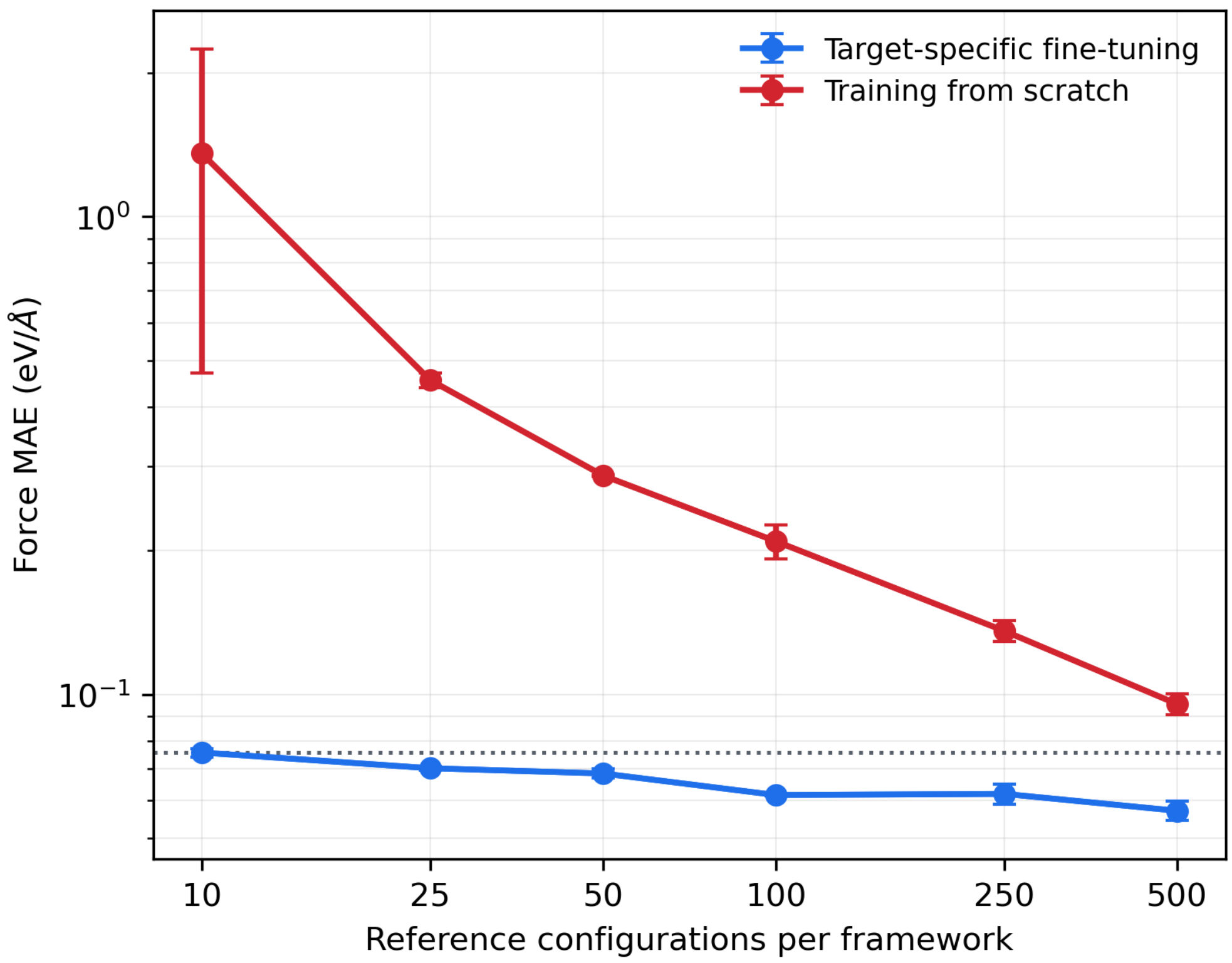

Figure S7. Learning curves for target-specific fine-tuning and training from scratch as a function of the number of reference configurations per framework. For each training-set size, three independently selected target-specific subsets were used. For each seed, the force MAE was first averaged over the four target ZIF systems. Symbols represent the mean over the three independent seeds, and error bars represent the corresponding standard deviation. The dotted horizontal line indicates the force MAE of the pretrained foundational MLP before target-specific fine-tuning.

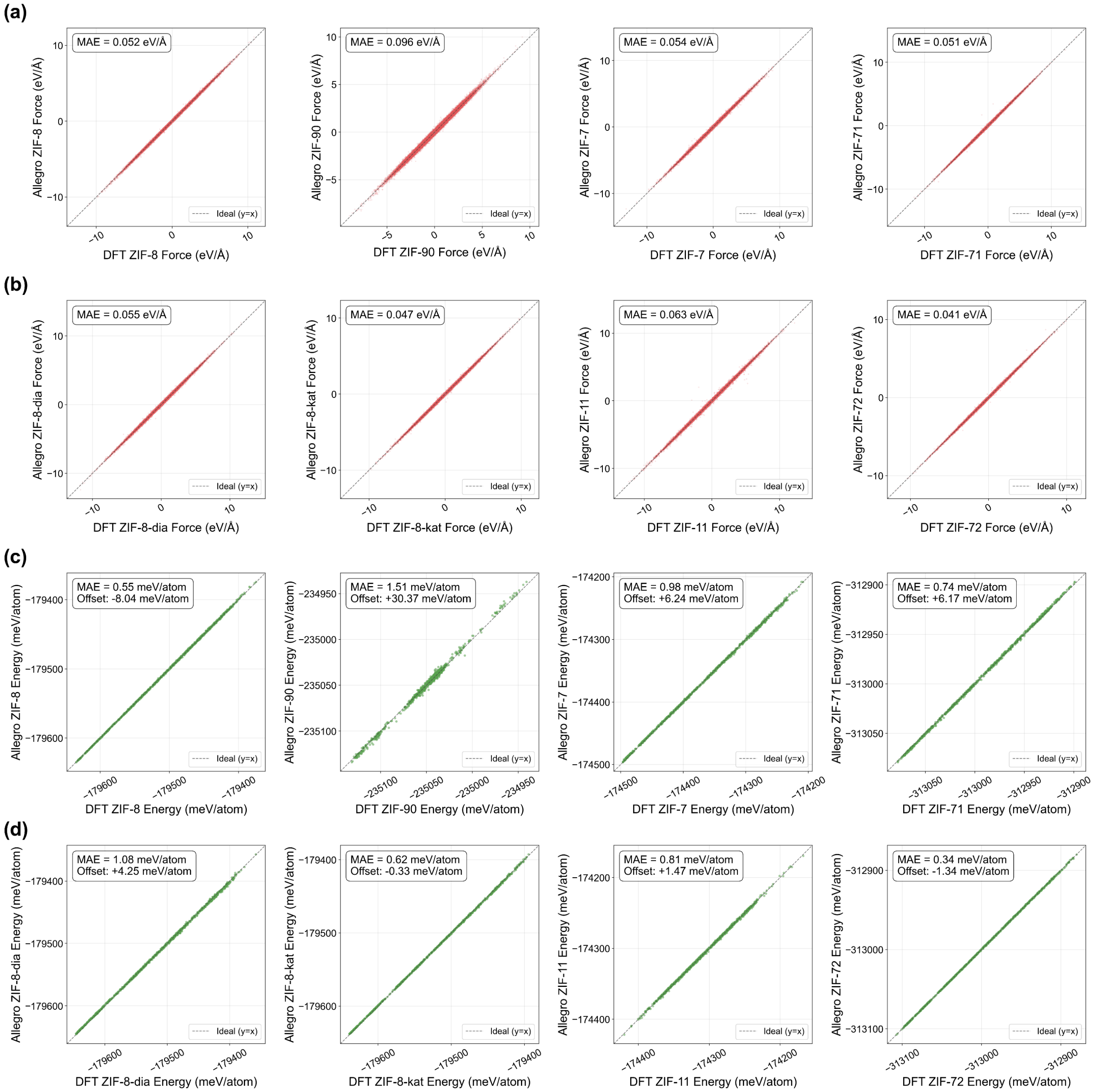

Figure S8. Force parity plots for the transfer-learned Zn-coordinated ZIF MLP showing (a) the pretraining frameworks and (b) the target frameworks. Atomic energy parity plots for the transfer-learned Zn-coordinated ZIF MLP showing (c) the pretraining frameworks and (d) the target frameworks.

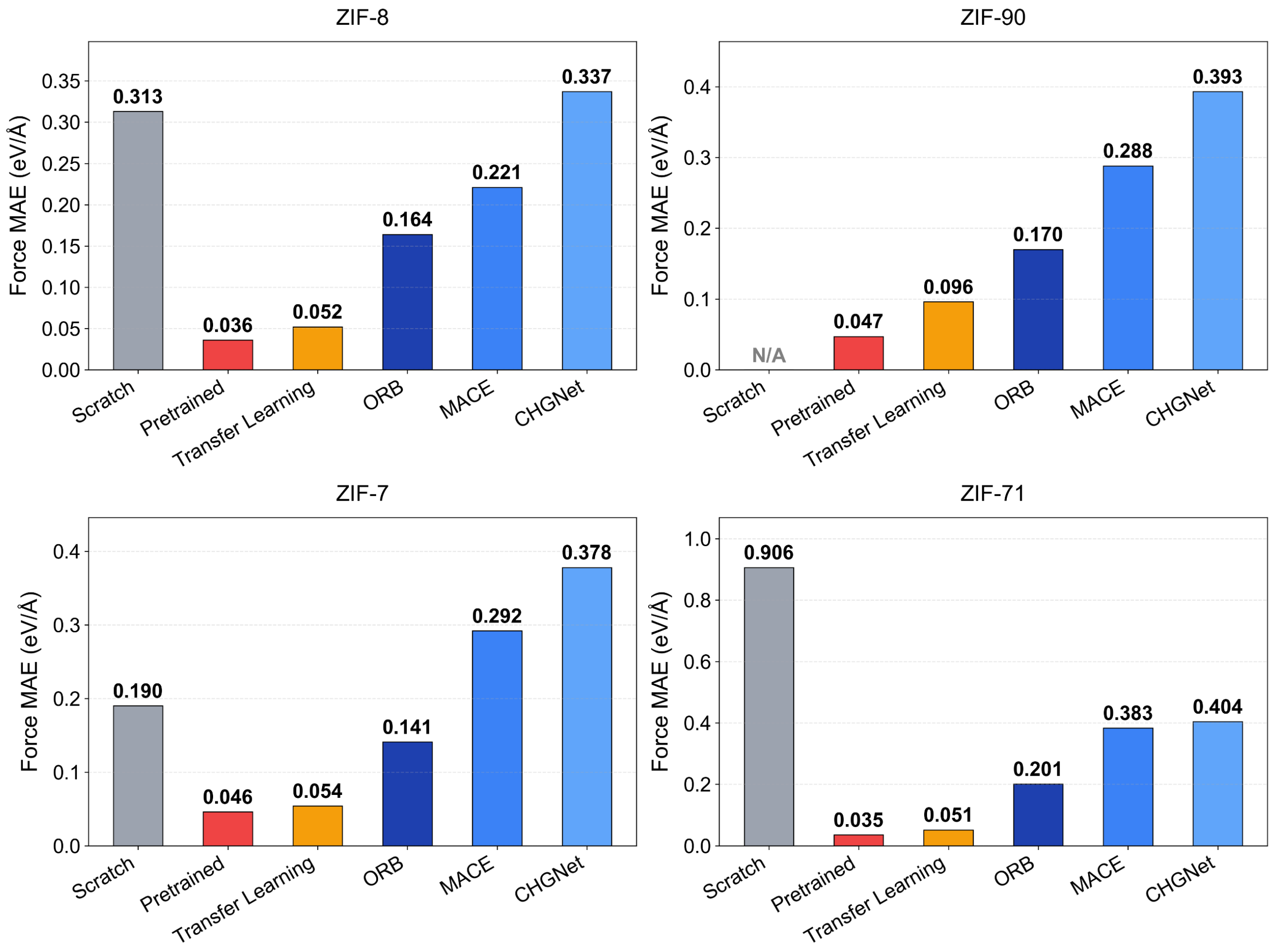

Figure S9. Comparison of force prediction accuracy obtained using Scratch, Pretrained, Transfer Learning, and foundation-model approaches for Zn-coordinated ZIF frameworks included in the transferable training dataset. Force mean absolute errors (MAEs, eV/Å) are reported for ZIF-8, ZIF-90, ZIF-7, and ZIF-71. Scratch models are trained using only 100 framework-specific configurations, Pretrained models correspond to the transferable Allegro potential, and Transfer Learning models are obtained by fine-tuning the pretrained model using the same 100 configurations. ORB, MACE, and CHGNet are included for comparison. Numerical values are summarized in **Table S5**.

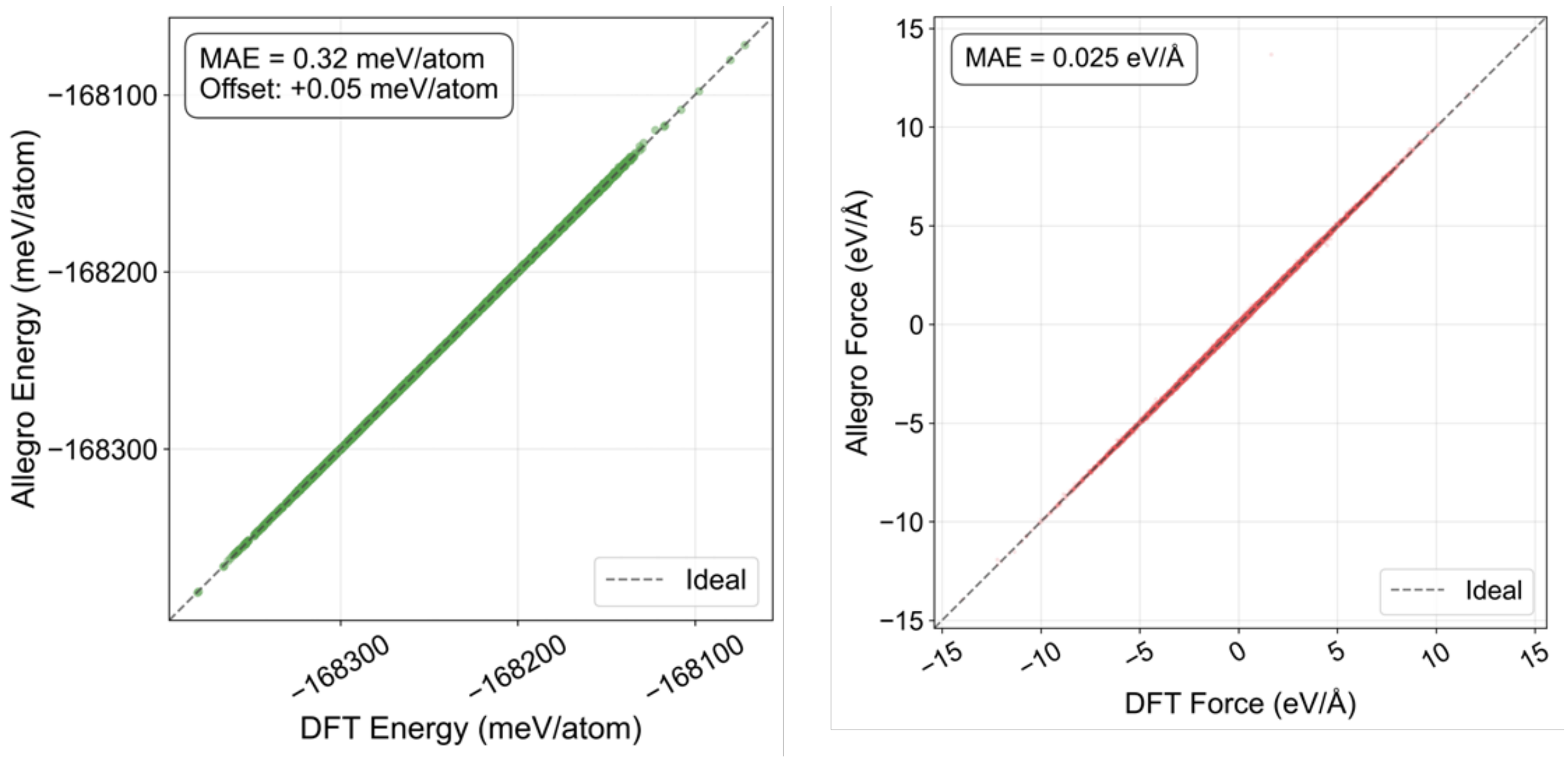

Figure S10. Validation of the methane–ZIF-8 MLP employed for methane-induced gate-opening simulations. Parity plots comparing predicted per-atom energies (left) and forces (right) with DFT reference values.

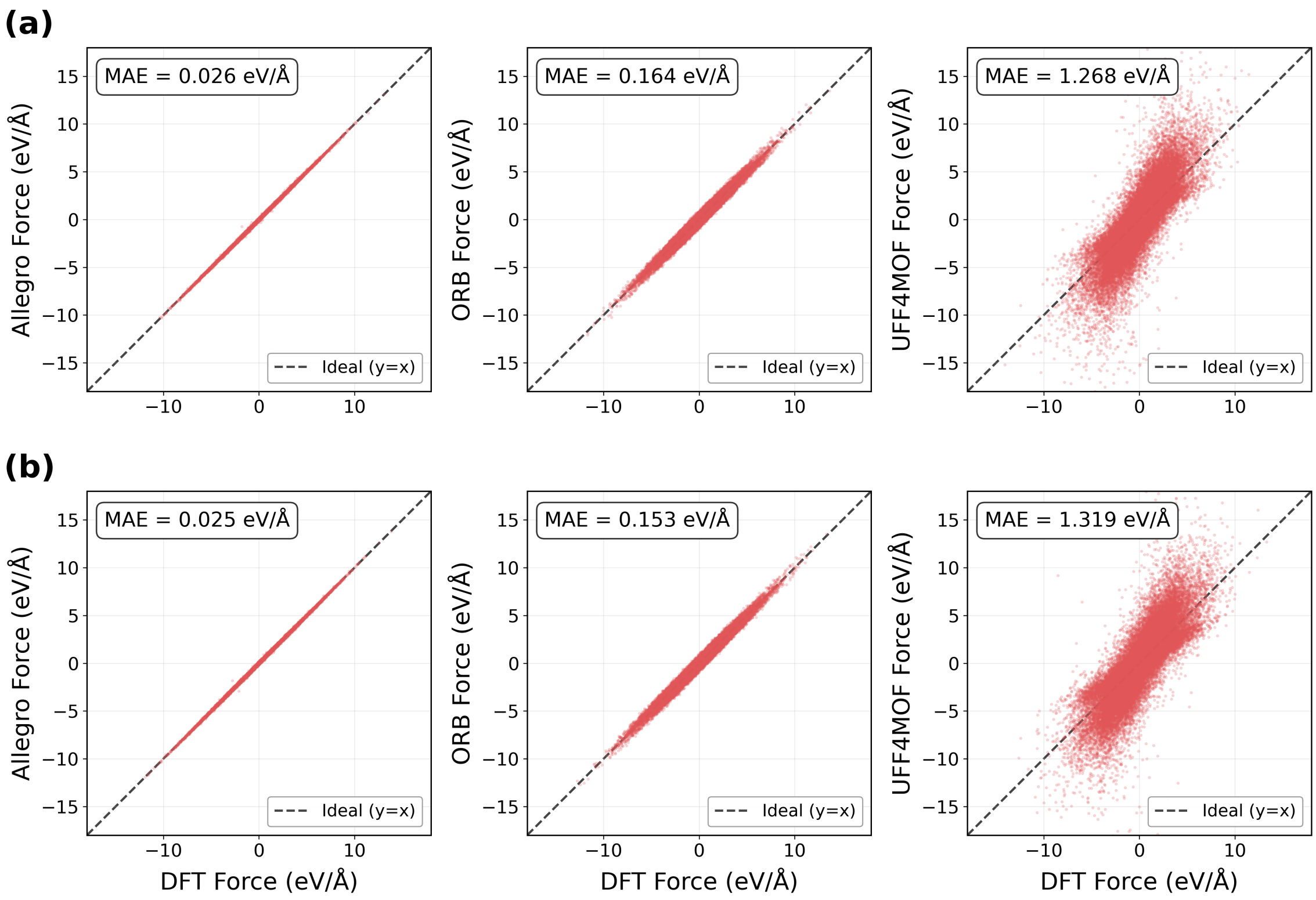

Figure S11. Force parity plots comparing the present Allegro MLP, ORB, and UFF4MOF against DFT reference forces for (a) pristine ZIF-8 and (b) methane-loaded ZIF-8 configurations. The force MAEs (eV/Å) are indicated for each model.

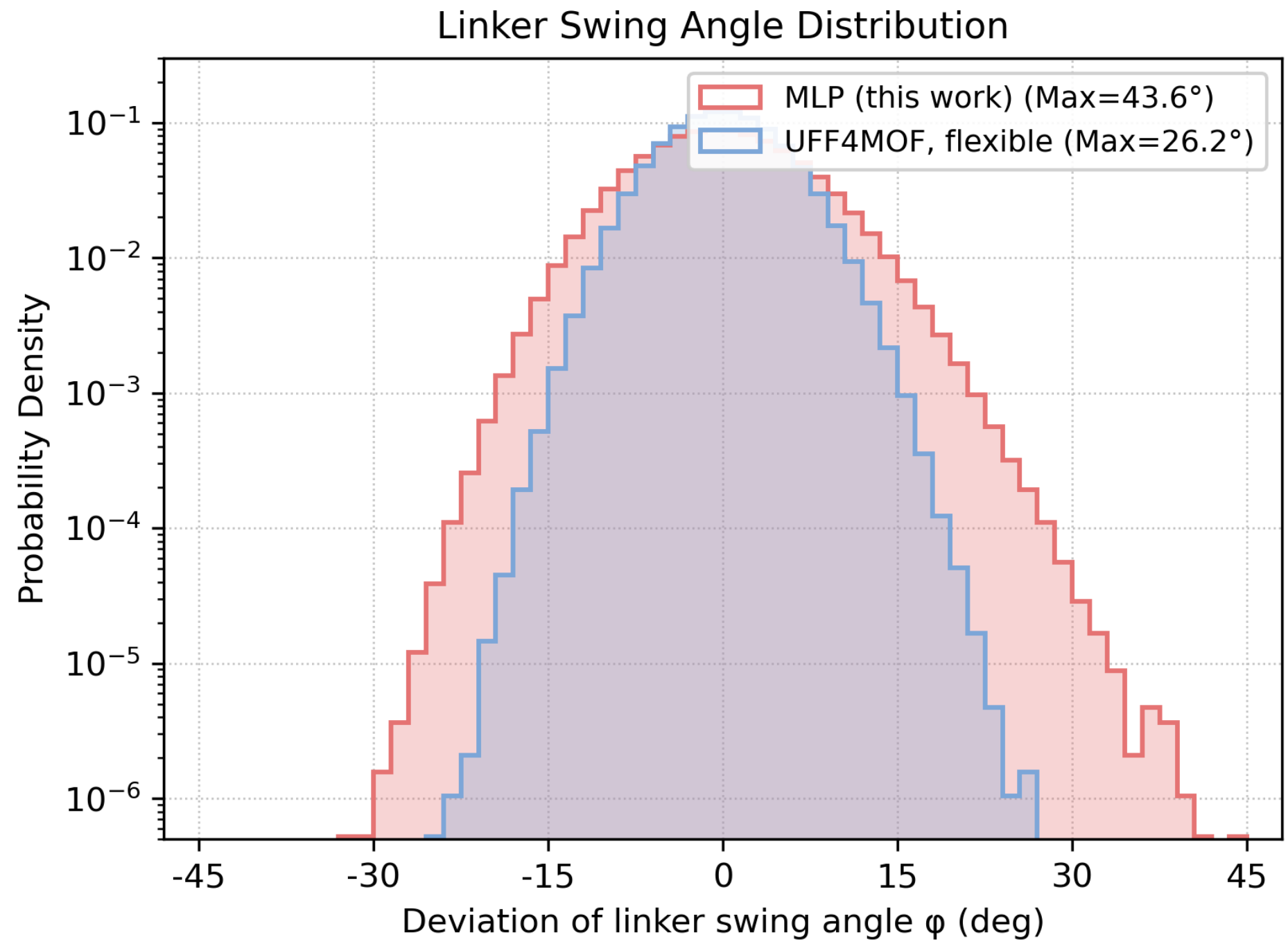

Figure S12. Comparison of linker swing-angle distributions obtained from MLP and flexible-framework UFF4MOF MD simulations of methane-loaded ZIF-8 at 200 K and 1 bar. Both simulations were performed using the same methane loading and simulation-cell conditions. The MLP exhibits a broader swing-angle distribution with more pronounced large-amplitude linker fluctuations than UFF4MOF, while both distributions remain centered near the equilibrium orientation.

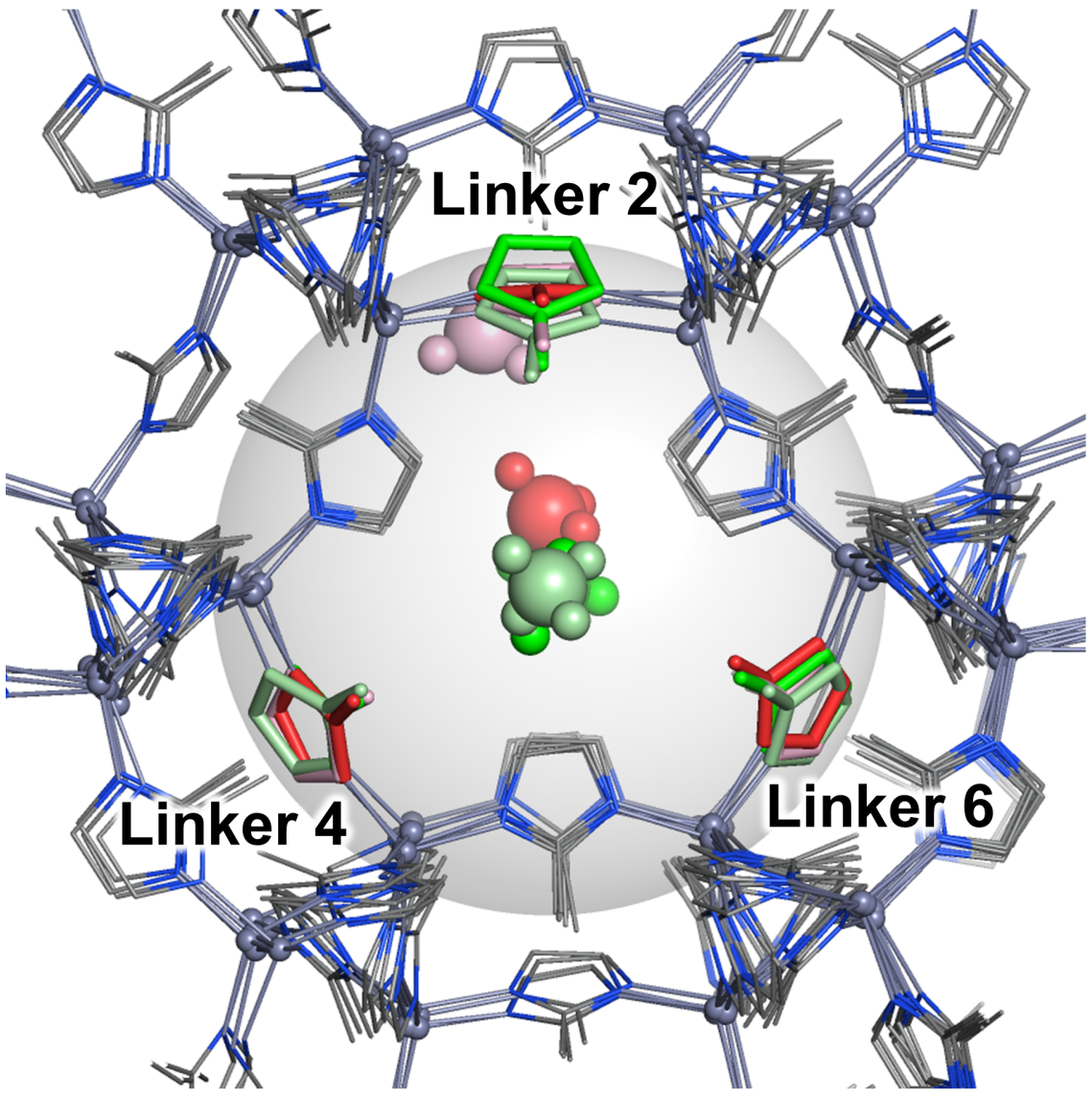

Figure S13. Identification of Linkers 2, 4, and 6 surrounding the ZIF-8 pore window. These linkers are selected for analysis because their methyl groups are oriented toward the pore window and directly influence the steric environment encountered by the permeating methane molecule.

3. Additional Tables referred in the main text

Table S1. Comparison of energy and force prediction errors for Allegro, ORB v3, MACE-medium, CHGNet, ReaxFF, and UFF4MOF on the ZIF-8 benchmark dataset. All values are reported relative to DFT reference calculations.

Force Field	Per-atom energy MAE (meV/atom)	Force MAE (eV/Å)
Allegro (this work)	0.11	0.016
ORB v3	1.99	0.164
MACE-medium	9.05	0.221
CHGNet	15.66	0.337
ReaxFF	18.51	1.741
UFF4MOF	57.87	0.942

Table S2. Comparison of force prediction accuracy and computational performance among the TorchScript-deployed Allegro potential, ORB v3, MACE-medium, MACE-MP-MOF0, and CHGNet. Force MAEs, MD throughput (ns/day), and peak GPU memory consumption (VRAM) are evaluated on the same 276-atom ZIF-8 benchmark system.

Model	Force MAE (eV/Å)	ns/day	VRAM (MB)
Allegro (.pth)	0.016	4.679	384
ORB v3	0.164	1.395	1220
MACE-medium	0.221	1.134	1240
MACE-MP-MOF0	0.092	1.094	1324
CHGNet	0.337	0.609	2709

Table S3. Comparison of predictive accuracy obtained using SOAP-based structural selection and random sampling at different training set sizes for ZIF-8 and the unseen ZIF-8(dia) topology.

Sampling Strategy	N_{train}	ZIF-8			ZIF-8(dia)		
		**Energy** (meV/atom)	**Corr. Energy** (meV/atom)	**Force** (eV/Å)	**Energy** (meV/atom)	**Corr. Energy** (meV/atom)	**Force** (eV/Å)
Full	40000	0.24	0.11	0.016	9.09	0.62	0.040
SOAP	1000	0.28	0.27	0.031	7.11	0.82	0.058
Random	1000	0.28	0.28	0.033	8.07	0.88	0.066
Random	2000	0.22	0.22	0.027	9.69	0.8	0.058
Random	4000	0.22	0.19	0.025	9.72	0.78	0.056
Random	8000	0.27	0.15	0.021	7.96	0.77	0.050
Random	16000	0.17	0.11	0.016	5.64	0.6	0.042

Table S4. Comparison of predictive accuracy between the transferable Allegro MLP developed in this work and state-of-the-art foundation models across Zn-coordinated ZIF frameworks. Per-atom energy MAEs and force MAEs are evaluated on test datasets for the training frameworks (ZIF-8, ZIF-90, ZIF-7, and ZIF-71) and the unseen frameworks (ZIF-8(dia), ZIF-8(kat), ZIF-11, and ZIF-72).

		Per-atom energy MAE (meV/atom)				Force MAE (eV/Å)			
		Allegro	ORB	MACE	CHGNet	Allegro	ORB	MACE	CHGNet
Training frameworks	ZIF-8	0.32	1.99	9.05	15.66	0.036	0.164	0.221	0.337
	ZIF-90	0.46	2.14	6.27	8.41	0.047	0.17	0.288	0.393
	ZIF-7	0.62	1.86	14.45	22.23	0.046	0.141	0.292	0.378
	ZIF-71	0.27	2.56	9.54	14.27	0.035	0.201	0.383	0.404
Unseen frameworks	ZIF-8 (dia)	1.9	2.03	10.43	19.72	0.088	0.141	0.217	0.327
	ZIF-8 (kat)	0.88	1.5	8.99	16.39	0.057	0.149	0.214	0.322
	ZIF-11	3.54	2.5	8.32	11.11	0.095	0.152	0.312	0.398
	ZIF-72	0.67	2.16	10.92	17.49	0.046	0.196	0.373	0.395

Table S5. Comparison of predictive performance obtained using Scratch, Pretrained, and Transfer Learning training strategies for Zn-coordinated ZIF frameworks. Per-atom energy MAEs and force MAEs are reported for both frameworks included in the pretraining dataset (ZIF-8, ZIF-90, ZIF-7, and ZIF-71) and target frameworks used for model specialization (ZIF-8(dia), ZIF-8(kat), ZIF-11, and ZIF-72).

		Per-atom energy MAE (meV/atom)			Force MAE (eV/Å)		
		Scratch	Pretrained	Transfer learning	Scratch	Pretrained	Transfer learning
Training frameworks	ZIF-8	9.87	0.32	0.55	0.313	0.036	0.052
	ZIF-90	N/A	0.46	1.51	N/A	0.047	0.096
	ZIF-7	5.79	0.62	0.98	0.190	0.046	0.054
	ZIF-71	43.54	0.27	0.74	0.906	0.035	0.051
Target frameworks	ZIF-8 (dia)	3.04	1.90	1.08	0.145	0.088	0.055
	ZIF-8 (kat)	2.72	0.88	0.62	0.142	0.057	0.047
	ZIF-11	5.06	3.54	0.81	0.194	0.095	0.063
	ZIF-72	2.64	0.67	0.34	0.210	0.046	0.041

4. References

(1) Kühne, T. D.; Iannuzzi, M.; Del Ben, M.; Rybkin, V. V.; Seewald, P.; Stein, F.; Laino, T.; Khaliullin, R. Z.; Schütt, O.; Schiffmann, F.; Golze, D.; Wilhelm, J.; Chulkov, S.; Bani-Hashemian, M. H.; Weber, V.; Borštnik, U.; Taillefumier, M.; Jakobovits, A. S.; Lazzaro, A.; Pabst, H., et al., CP2K: An electronic structure and molecular dynamics software package - Quickstep: Efficient and accurate electronic structure calculations. *J. Chem. Phys.* **2020,** *152*, 194103.

(2) Castel, N.; André, D.; Edwards, C.; Evans, J. D.; Coudert, F.-X., Machine learning interatomic potentials for amorphous zeolitic imidazolate frameworks. *Digit. Discov.* **2024,** *3*, 355-368.

(3) Bartók, A. P.; Kondor, R.; Csányi, G., On representing chemical environments. *Phys. Rev. B* **2013,** *87*, 184115.

(4) Musaelian, A.; Batzner, S.; Johansson, A.; Sun, L.; Owen, C. J.; Kornbluth, M.; Kozinsky, B., Learning local equivariant representations for large-scale atomistic dynamics. *Nat. Commun.* **2023,** *14*, 579.

(5) Tan, C. W.; Descoteaux, M. L.; Kotak, M.; de Miranda Nascimento, G.; Kavanagh, S. R.; Zichi, L.; Wang, M.; Saluja, A.; Hu, Y. R.; Smidt, T.; Johansson, A.; Witt, W. C.; Kozinsky, B.; Musaelian, A., High-performance training and inference for deep equivariant interatomic potentials. *Digit. Discov.* **2026,** *5*, 1558-1567.
